# Critical appraisal and external validation of a prognostic model for survival of people living with HIV/AIDS who underwent antiretroviral therapy

**DOI:** 10.1186/s41512-020-00088-x

**Published:** 2020-11-25

**Authors:** Junfeng Wang, Tanwei Yuan, Xuemei Ling, Quanmin Li, Xiaoping Tang, Weiping Cai, Huachun Zou, Linghua Li

**Affiliations:** 1 Julius Center for Health Sciences and Primary Care, University Medical Center Utrecht, Utrecht University, Universiteitsweg 100, 3584 CG Utrecht, the Netherlands; 2 School of Public Health (Shenzhen), Sun Yat-sen University, Shenzhen, China; 3 Guangzhou Eighth People’s Hospital, Guangzhou Medical University, No.627 Dongfeng Dong Road, Guangzhou, 510060 Guangdong China; 4 Kirby Institute, the University of New South Wales, Sydney, Australia

**Keywords:** Prognostic model, External validation, People living with HIV/AIDS, Survival

## Abstract

**Background:**

HIV/AIDS remains a leading cause of death worldwide. Recently, a model has been developed in Wenzhou, China, to predict the survival of people living with HIV/AIDS (PLWHA) who underwent antiretroviral therapy (ART). We aimed to evaluate the methodological quality and validate the model in an external population-based cohort.

**Methods:**

Prediction Model Risk of Bias Assessment Tool (PROBAST) was used to assess the risk of bias of the Wenzhou model. Data were from the National Free Antiretroviral Treatment Program database. We included PLWHA treated between February 2004 and December 2019 in a tertiary hospital in Guangzhou city, China. The endpoint was all-cause deaths and assessed until January 2020. We assessed the discrimination performance of the model by Harrell’s overall C-statistics and time-dependent C-statistics and calibration by comparing observed survival probabilities estimated with the Kaplan–Meier method versus predicted survival probabilities. To assess the potential prediction value of age and gender which were precluded in developing the Wenzhou model, we compared the discriminative ability of the original model with an extended model added with age and gender.

**Results:**

Based on PROBAST, the Wenzhou model was rated as high risk of bias in three out of the four domains (selection of participants, definition of outcome, and methods for statistical analysis) mainly because of the misuse of nested case–control design and propensity score matching. In the external validation analysis, 16758 patients were included, among whom 743 patients died (mortality rate 11.41 per 1000 person-years) during follow-up (median 3.41 years, interquartile range 1.64–5.62). The predictor of HIV viral load was missing in 14361 patients (85.7%). The discriminative ability of the Wenzhou model decreased in the external dataset, with the Harrell’s overall C-statistics being 0.76, and time-dependent C-statistics dropping from 0.81 at 6 months to 0.48 at 10 years after ART initiation. The model consistently underestimated the survival, and the level was 6.23%, 10.02%, and 14.82% at 1, 2, and 3 years after ART initiation, respectively. The overall and time-dependent discriminative ability of the model improved after adding age and gender to the original model.

**Conclusion:**

The Wenzhou prognostic model is at high risk of bias in model development, with inadequate model performance in external validation. Thereby, we could not confirm the validity and extended utility of the Wenzhou model. Future prediction model development and validation studies need to comply with the methodological standards and guidelines specifically developed for prediction models.

**Supplementary Information:**

**Supplementary information** accompanies this paper at 10.1186/s41512-020-00088-x.

## Introduction

Despite substantial progress made in expanding antiretroviral therapy (ART) coverage and reducing overall HIV-related mortality over the past decade, HIV/AIDS remains a huge health burden worldwide [1]. Globally, the number of people living with HIV/AIDS (PLWHA) has increased from 8.74 million in 1990 to 36.8 million in 2017 [1], and HIV/AIDS remains the leading cause of death for nearly 1 million people every year [1–3]. This calls for continuous efforts and health resources for HIV/AIDS treatment and disease management.

An ideal prognostic model for PLWHA would be crucial in optimizing HIV care and treatment tailored to each patient, which could improve treatment outcomes and help the rational allocation of limited health resources [4]. Thus, several prognostic models and risk scoring systems based on datasets from Europe and North America have been developed to predict treatment outcomes (e.g., mortality, HIV virological failure) of PLWHA who underwent ART [5–9], and a few of them have been updated [10] and externally validated [11, 12].

Recently, a nested case–control study including 750 PLWHA from Wenzhou, China, developed and comprehensively validated a prognostic model for predicting the HIV-related death of PLWHA receiving ART (herein after uniformly referred to as the Wenzhou model) and first developed a simple and intuitive nomogram to help its application among healthcare providers [13]. This is the first prognostic model for PLWHA developed in the Western Pacific region. This model incorporates three baseline parameters: hemoglobin, HIV viral load, and CD4^+^ cell counts, which could stratify patients into three risk groups depending on the overall prognostic scores calculated by the nomogram [13]. In the random split internal validation, the model showed exceptionally excellent discriminative power, predictive accuracy, and clinical utility [13].

However, the methodology used to develop and validate the prognostic model in the Wenzhou study needs to be critically assessed, and the promising performance of the model ought to be validated in an independent sample of patients for its generalization and clinical application. External validation is indispensable to establish the transportability and general applicability of a model [14, 15]. Various clinical practice guidelines recommend only those prognostic models that have repeatedly demonstrated good predictive accuracy in multiple validation studies could be incorporated in clinical practice [14, 16]. The inadequacy of external validation could largely explain why so far none of these existing prognostic models for PLWHA has been widely implemented or used in clinical practice [15].

Guangdong is the most populous province in China, with a population of nearly 113.46 million in 2018 [17]. A total of 81641 cumulated HIV cases had been reported in Guangdong by 2017, of whom 2100 had died [18]. In this study, our first aim was to use Prediction model Risk Of Bias Assessment Tool (PROBAST) [19, 20] to formally assess the methodological quality of the Wenzhou model and, next to it, externally validate the Wenzhou model in a large population-based cohort of PLWHA from Guangzhou, the capital city of Guangdong province, China.

## Methods

### Study design and participants

This retrospective observational cohort study used data retrieved from the National Free Antiretroviral Treatment Program database. This database, which is managed by the National Center for AIDS/STD Control and Prevention, China Center for Disease Control and Prevention (China CDC), has been described elsewhere [21]. Each hospital has access to data for its jurisdiction. We included PLWHA treated in the Guangzhou Eighth People’s Hospital, a well-established tertiary infectious diseases hospital, between 10 February 2004 and 5 December 2019, and data were collected from 10 February 2004 up to 1 January 2020. According to the inclusion criteria used in the Wenzhou study [13], we included patients who initiated a combination ART regimen contained at least three drugs in the center, above 15 years of age, and had at least one follow-up record.

Baseline and follow-up information was all assessed based on standardized case report forms that were completed by local healthcare providers and then uploaded to the central database. Details on data collection could be found elsewhere [21]. Information on the three predictors of the Wenzhou model (i.e., hemoglobin, HIV viral load, and CD4^+^ cell counts) was assessed in the central laboratory of the center by trained technicians within 1 week before ART initiation in the center. Other baseline information included clinical data (age, gender, marital status, residence, route of HIV acquisition, WHO clinical staging of HIV disease, tuberculosis infection status, body weight, height), laboratory parameters (CD8 cell counts, HBsAg status, white blood cell count, platelet, creatinine, triglyceride, total cholesterol, plasma glucose, plasma glucose, aspartate transaminase, alanine aminotransferase, total bilirubin), and initial ART regimen. Information on clinical and laboratory characteristics, last follow-up date, or the date of clinical outcomes was collected at scheduled follow-up visits (0.5, 1, 2, and 3 months after ART initiation and every 3 months thereafter). Information on death was determined via standardized follow-up case report forms.

### Methodology quality assessment

We assessed the risk of bias of the Wenzhou prognostic model based on PROBAST [19, 20]. PROBAST was originally designed for systematic reviews, but it can also be used in critical appraisal of the methodological quality of prediction models [19, 20]. This instrument assesses the risk of bias of prediction model studies in four broad domains: participants (2 signaling questions [SQ]), predictors (3 SQ), outcome (6 SQ), and analysis (9 SQ). Each domain is rated as high (the answer to any of the SQ in that domain is “No” or “Probably no”), low (the answer to all SQ is “Yes” or “Probably yes”), or unclear (relevant information is missing for some of the signaling questions, and the answer to all remaining questions is “Yes” or “Probably yes”) risk of bias [19, 20]. The rationale for rating each criterion was recorded. Two authors (JW and TY) independently assessed the risk of bias of the Wenzhou study, and the agreement of two raters was measured by the percentage of agreement and Cohen’s kappa. Any disagreement was resolved through discussion. Whenever necessary, a senior author (HZ) made the final decision.

### External validation

#### Statistical analysis

All statistical analyses were performed using R version 3.5.1, and R code used for the external validation can be found in the supplement. We conducted and reported this study according to recommendations in the Transparent Reporting of a multivariable prediction model for Individual Prognosis or Diagnosis statement (TRIPOD) [4, 15], and the completed checklist could be found in supplement table 1.

The sample size of the study was determined by all available data on the database of the center during the study period. The endpoint was all-cause deaths, and survival time was measured as the date of ART initiation in the center to date of death, date of the last follow-up visit, or 1 January 2020, whichever came first. Median follow-up time was computed using a reverse Kaplan–Meier method [4]. Baseline characteristics of patients were presented as count (percentage) for categorical variables and median (interquartile range) for continuous variables. Study information, baseline characteristics, and outcomes of this study were compared with that of the Wenzhou study.

We assessed the predictive performance of the Wenzhou model by examining the measures of discrimination and calibration [22]. Discrimination was assessed by Harrell’s overall C-statistics [23] with R package “compareC” [24] as well as time-dependent C-statistics [25, 26] with R package “riskRegression” [27]. A C-statistics of 0.5 represents no predictive discrimination and 1 represents perfect discrimination. Calibration was assessed with the calibration curve plot by comparing the observed survival probability estimated with the Kaplan–Meier method versus the predicted survival probability.

In addition, age and gender were precluded as candidate predictors for developing the Wenzhou model as the model development study used a nested case–control design with age and gender being used for matching. To assess the potential prediction value of age and gender, we extended the model by adding these two variables to the original Wenzhou model. In order to avoid the overestimation of the added prediction value, we did not re-fit the extended model in our external validation data, and the coefficients of the two variables were obtained from the literature review. The reported effect size (i.e., hazard ratio) of age and gender was log-transformed to obtain the regression coefficients for the extended model. The predictive ability of age and gender was investigated by comparing the C-statistics of the extended model with the Wenzhou model.

We used multiple imputation to impute (50 times) missing predictor values with R package “MICE” [28]. Variables used in the imputation model included all the predictors, age, gender, marital status, residence, route of HIV acquisition, WHO clinical staging of HIV disease, tuberculosis infection status, body weight, height, CD8 cell counts, HBsAg status, white blood cell count, platelet, creatinine, triglyceride, total cholesterol, plasma glucose, plasma glucose, aspartate transaminase, alanine aminotransferase, total bilirubin, initial ART regimen, and the outcome (i.e., the Nelson–Aalen estimator of the cumulative baseline hazard, and the outcome indicator) [29, 30]. Given that laboratory measurements (e.g., white blood cell count) can only have positive values and were possibly skewed, we applied a logarithmic transformation to all measured laboratory indexes to achieve normalization before they were included in the imputation model. The multiple imputation generated 50 plausible imputed datasets to account for the uncertainty associated with missing values. All the 50 imputed datasets were analyzed in parallel as if complete cases without missing predictor values, and at last, results obtained from each dataset were combined with Rubin’s rule [31, 32]. We did a descriptive analysis for predictor values before and after multiple imputation. The analysis of the imputation datasets was our main analysis with results being reported in this report, and complete case analysis was performed as sensitivity analysis with results being included in the supplement. Given that the percentage of missing values for HIV viral load in our dataset was high (86%) and 96% of the non-missing values were above 1000 copies/mL, we did two additional sensitivity analyses (1) assuming all the missing values for HIV viral load were < 200 copies/mL and (2) assuming missing values for HIV viral load had the same distribution as reported in the Wenzhou study. The category of imputed HIV viral load was determined by the quantile of the imputed value according to the distribution of HIV viral load reported in the Wenzhou study.

#### Prediction calculation

To calculate the predicted probability with the Wenzhou model in the external validation dataset, we extracted the model parameters (i.e., coefficients and baseline survival) from the nomogram of the Wenzhou model with GetData Graph Digitzer version 2.26. The corresponding author of the Wenzhou study was contacted by email if additional information was needed.

We first calculated the prognostic index (PI, i.e., the linear predictor of the model) for each patient (*i*) using the following formula:

$$ {\mathrm{PI}}_i={\mathrm{Coefficient}}_{\mathrm{hemoglobin}}\times {\mathrm{Hemoglobin}}_i+\kern0.5em {\mathrm{Coefficient}}_{\mathrm{CD}4\ \mathrm{cell}\ \mathrm{count}}\times \mathrm{CD}4\ {\mathrm{cell}\ \mathrm{count}}_i+{\mathrm{Coefficient}}_{\mathrm{HIV}\ \mathrm{viral}\ \mathrm{load}}\times \mathrm{HIV}\ {\mathrm{viral}\ \mathrm{load}}_i $$

Based on the coefficients extracted from the nomogram:

$$ {\mathrm{PI}}_i=-0.005580907\times \mathrm{CD}4\kern0.5em \mathrm{cell}\kern0.5em {\mathrm{count}}_i-0.005368102\times {\mathrm{hemoglobin}}_i\kern0.5em + $$

$$ 1.019669556\kern0.5em \left[\mathrm{if}\kern0.5em \mathrm{HIV}\kern0.5em \mathrm{viral}\kern0.5em {\mathrm{load}}_i\kern0.5em \mathrm{is}\kern0.5em \mathrm{within}\kern0.5em 200-1000\right]+2.608969326\kern0.5em \left[\mathrm{if}\kern0.5em \mathrm{HIV}\kern0.5em \mathrm{viral}\kern0.5em {\mathrm{load}}_i\ge 1000\right] $$

We then calculated the predicted survival probability at *t* year (*t* = 1, 2, and 3) after ART initiation for each patient (*i*) using the following formula:

$$ \hat{S_i}(t)=\hat{S_0}{(t)}^{\exp \left({\mathrm{PI}}_i\right)} $$

where $\hat{S_0}(t)$ is the baseline survival at *t* year, PI_*i*_ is the prognostic index of patient *i*, and exp stands for exponential function.

Based on the extracted baseline survival probabilities, the 1-year, 2-year, and 3-year survival probabilities for patient (*i*) can be calculated as:

$$ \hat{S_i}(1)={0.980222074}^{\exp \left({\mathrm{PI}}_i\right)} $$

$$ \hat{S_i}(2)={0.972736744}^{\exp \left({\mathrm{PI}}_i\right)} $$

$$ \hat{S_i}(3)={0.964896148}^{\exp \left({\mathrm{PI}}_i\right)} $$

To create risk groups, we also extracted the linear relation between PI and risk score from the nomogram, and the risk score for each patient (*i*) was calculated by:

$$ {\mathrm{Risk}\ \mathrm{score}}_i=108.3333333+19.90914787\times {\mathrm{PI}}_i $$

To assess the added prediction value of age and gender, an extended formula which added age and gender was used, where the coefficients of these two variables were based on evidence from literatures [33–35].

$$ \mathrm{PI}\_{\mathrm{extended}}_i={PI}_i+0.2382292\ \left[\mathrm{if}\ {\mathrm{age}}_i\ \mathrm{is}\ \mathrm{within}\ 40-60\right]+0.5866749\ \left[\mathrm{if}\ {\mathrm{age}}_i\ge 60\right]-0.3566749\ \left[\mathrm{if}\ {\mathrm{gender}}_i=\mathrm{female}\right] $$

## Results

### Characteristics of patients

**Fig. 1:**
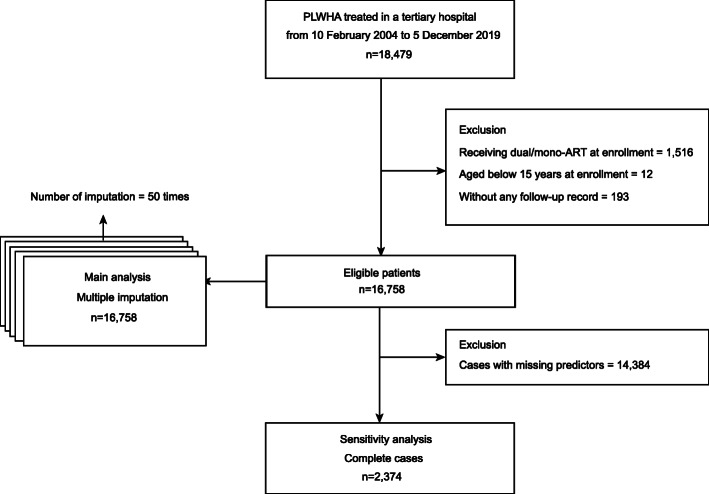
Flow chart of the selection of patients. PLWHA people living with HIV/AIDS; ART antiretroviral therapy

Data were obtained from 18479 patients with HIV, of whom 16758 met the inclusion criteria and were included in our main analysis after multiple imputation of missing predictors (Fig. 1). Additionally, we also did a sensitivity analysis for the 2374 complete cases after excluding 14384 patients with missing predictors. The cumulative incidence curve of complete cases and that of cases with missing predictors were comparable (supplement figure 1). Descriptive analysis of the three predictors before and after multiple imputation can be found in supplement table 2. The median follow-up for the 16758 participants was 3.41 years (IQR [interquartile range] 1.64–5.62). A total of 743 (4.43%) participants died from any cause during 65037 person-years of follow-up (mortality per 1000 person-years, 95% CI [confidence interval] 11.42, 10.62–12.28). Cumulative mortality rates of all-cause death at 3, 5, 10, and 15 years after ART initiation in the center were 3.72%, 5.11%, 8.96%, and 11.35%, respectively.

Table 1 compares the characteristics and outcomes of the participants in the cohort to develop the Wenzhou model and that in this external validation study.

**Table 1:** Comparison of participants characteristics and outcomes in derivation and external validation cohorts*

Characteristics	Derivation cohort of the Wenzhou model [13]		External validation cohort	
	Total (***N*** = 525)	Missing values (***n*** (%))	Total (***N*** = 16758)	Missing values (***n*** (%))
**Study information**				
Geographic location	East China	..	South China	..
Data source	AIDS Prevention and Control Information System in Wenzhou city	..	National Free Antiretroviral Treatment Program database in a tertiary hospital in Guangzhou city	..
Follow-up duration	January 2006 to December 2018	..	February 2004 to 1 January 2020	..
**Variables in the prognostic model of the Wenzhou study**				
Viral load, copies/mL		0 (0.0)		14361 (85.7)
< 200	413 (78.7)	..	39 (1.6)	..
200–1000	16 (3.0)	..	57 (2.4)	..
≥ 1000	96 (18.3)	..	2301 (96.0)	..
CD4^+^ cell count, cells/μL	208.3 (86.0, 328.0)	..	209.00 (72.0, 319.0)	173 (1.1)
Hemoglobin, g/L	136.7 (118.0, 149.0)	..	137.00 (116.0, 150.0)	237 (1.4)
**Variables used for matching by the Wenzhou study**				
Age, year	49.7 (37.3, 63.5)	0 (0.0)	34.3 (27.2, 44.0)	0 (0.0)
Gender		0 (0.0)		0 (0.0)
Men	436 (83.0)	..	13662 (81.5)	..
Women	89 (17.0)	..	3096 (18.5)	..
**Other variables in the Wenzhou study**^†^				
Hepatitis B virus		0 (0.0)		3152 (18.8)
Positive	68 (13.0)	..	1799 (13.2)	..
Negative	457 (87.0)	..	11807 (86.8)	..
Tuberculosis		13 (2.5)		17 (0.1)
Yes	24 (4.6)	..	1097 (6.6)	..
No	488 (93.0)	..	15644 (93.5)	..
WHO stage		0 (0.0)		17 (0.1)
I	265 (50.5)	..	417 (2.5)	..
II	53 (10.1)	..	392 (2.3)	..
III	157 (29.9)	..	15535 (92.8)	..
IV	50 (9.5)		397 (2.4)	..
Infection pathway		0 (0.0)		692 (4.1)
NMHR	304 (57.9)	..	7473 (46.5)	..
MSM	158 (30.1)	..	7271 (45.3)	..
Others	63 (12.0)	..	1322 (8.2)	..
Participant category		0 (0.0)		5 (0.03)
Fixed population	407 (77.5)		10966 (65.4)	..
Floating population	118 (22.5)		5787 (34.5)	..
Marital status		0 (0.0)		105 (0.6)
Married	277 (52.8)	..	7973 (47.9)	..
Unmarried	248 (47.2)	..	8680 (52.1)	..
Body mass index, kg/m^2‡^	21.3 (19.3, 23.7)	0 (0.0)	20.76 (19.0, 22.9)	2667 (15.9)
CD8 cell count, cells/μL	861.0 (535.0, 1272.0)	0 (0.0)	786.00 (521.0, 1121.0)	416 (2.5)
White blood cell, 10^9^/L	5.3 (4.2, 6.8)	0 (0.0)	5.1 (4.1, 6.4)	220 (1.3)
Platelet, 10^9^/L	186.0 (144.0, 224.0)	0 (0.0)	200.0 (160.0, 242.0)	230 (1.4)
Creatinine, μmol/L	70.0 (58.6, 81.0)	0 (0.0)	74.0 (63.8, 84.0)	516 (3.1)
Triglyceride, mmol/L	1.5 (1.0, 2.4)	0 (0.0)	1.3 (0.9, 1.8)	2555 (15.3)
Total cholesterol, mmol/L	4.1 ± 0.9	0 (0.0)	4.0 (3.4, 4.6)	2559 (15.3)
Plasma glucose, mmol/L	5.3 (4.7, 6.6)	0 (0.0)	5.2 (4.7, 5.7)	1449 (8.7)
Aspartate transaminase, U/L	25.0 (19.0, 34.0)	0 (0.0)	23.00 (19.00, 32.00)	249 (1.5)
Alanine aminotransferase, U/L	21.6 (15.0, 34.0)	0 (0.0)	23.00 (16.0, 36.0)	232 (1.4)
Total bilirubin, μmol/L	10.8 (7.7, 15.4)	0 (0.0)	9.73 (7.2, 13.0)	361 (2.2)
**Other variables in the validation cohort**				
ART initiation year^§^				0 (0.0)
2004-2007	..	..	575 (3.4)	..
2008-2011	..	..	2227 (13.3)	..
2012-2015	..	..	5810 (34.7)	..
2016-2019	..	..	8146 (48.6)	..
Baseline ART regimen				0 (0.0)
First-line^¶^	..	..	13286 (79.3)	..
Others	..	..	3472 (20.7)	..
**Outcomes**				
Endpoint	HIV-related mortality	..	All-cause mortality	..
Number of events	105	..	743	..
Mortality per 1000 person-years	73.1	..	11.4	..

*NMHR* non-marital heterosexual transmission, *MSM* men who have sex with men, *WHO* World Health Organization, *ART* antiretroviral therapy

*Categorical variables are presented as *n* (%), and continuous variables are presented as median (interquartile range)

^†^Occupation, history of sexually transmitted diseases other than HIV/AIDS, education level, disease stage, and origin of identification were also included in the Wenzhou study but were not included in this study because these data were unavailable in our database

^‡^Body mass index = body weight/height^2^

^§^Cut-off values of years were determined by changes in Chinese national guidelines for the treatment of HIV/AIDS regarding the threshold of CD4^+^ cell counts for initiating ART. Prior to 2007, HIV-infected patients with a CD4^+^ count ≤ 200 cells per μL or those who had been diagnosed with an AIDS-defining illness were eligible for ART initiation. The treatment initiation threshold was raised to 350 cells per μL in 2008 and then to 500 cells per μL in 2012. Since 2016, all PLWHA have been eligible for ART regardless of CD4^+^ count

^¶^First-line ART regimens consist of zidovudine/stavudine/tenofovir + lamivudine + nevirapine/efavirenz

Compared to the patients in the derivation cohort of the Wenzhou model, patients in our study were younger (median 34.3 vs 49.7) and were more clinically advanced when they initiated ART in the center (WHO stage III/IV 95.2% vs 39.4%), and a higher proportion of them had HIV viral load equal to or more than 1000 copies/mL (96.0% vs 18.3%). Missing values of most variables in our study were higher than those in the Wenzhou study, especially HIV viral load (85.7% vs 0.0%), though the sample size of our study was larger (16758 vs 525). Regarding outcomes, the assessed endpoint in our study was all-cause mortality, noticeably lower than the endpoint of HIV-related mortality in the Wenzhou study (11.4 vs 73.1 per 1000 person-years). Additionally, although the starting point of survival time defined in the Wenzhou study (i.e., receiving the first ART) differs from that in our study (i.e., starting ART in the center), up to 99.2% (16631/16758) of patients included in this study initiated their ART in the Guangzhou Eighth People’s Hospital.

### Methodology quality assessment

The degree of agreement between the two authors who independently assessed the risk of bias was moderate before discussion (agreement in 70% of all items, Cohen’s kappa = 0.476, supplement table 2), and all the disagreements were settled after discussion. Overall, according to the PROBAST, the Wenzhou model was rated as high risk of bias in three domains: participants, outcome, and analysis (Table 2). The answer to more than half (11/20, 55%) of the total SQ was “No” or “Probably no.” The high risk of bias was judged according to some specific issues in the study design and statistical analysis (see the rationale of rating in Table 1). We elaborated on the main issues as below.

**Table 2:** Quality assessment by prediction model risk of bias assessment tool

Question	Answer	Rationale
**Domain 1: Participants**		
1.1 Were appropriate data sources used, e.g., cohort, RCT or nested case–control study data?	No	Nested case–control without proper adjustment of the baseline hazard.
1.2 Were all inclusions and exclusions of participants appropriate?	Probably no	Having complete laboratory blood tests before receiving ART may lead to biased selection.
**Overall risk of bias of Domain 1**	**High risk of bias**	
**Domain 2: Predictors**		
2.1 Were predictors defined and assessed in a similar way for all participants?	Probably yes	Laboratory outcomes were obtained in a standardized manner, whereas self-reported data such as mode of HIV transmission might be subjected to bias from self-interpretation. Nevertheless, only laboratory outcomes were included in the final prognostic model.
2.2 Were predictor assessments made without knowledge of outcome data?	Yes	The outcome was death, and predictor data were collected at patients' enrollment.
2.3 Are all predictors available at the time the model is intended to be used?	Yes	All predictors (i.e., hemoglobin, CD4^+^ cell count, and HIV viral load) are routine laboratory assessment and easy to access.
**Overall risk of bias of Domain 2**	**Low risk of bias**	
**Domain 3: Outcome**		
3.1 Was the outcome determined appropriately?	Probably no	1. Determination of AIDS-related death was unclear, so misclassification of outcomes might be possible. 2. Given that loss to follow-up was not mentioned in the paper, participants who were lost to follow-up might be misclassified as being alive.
3.2 Was a pre-specified or standard outcome definition used?	No information	Definition of AIDS-related death was not provided.
3.3 Were predictors excluded from the outcome definition?	Yes	The outcome was death, which is objective.
3.4 Was the outcome defined and determined in a similar way for all participants?	No information	The authors did not provide any information regarding how AIDS-related death was determined and whether it varied from patients to patients.
3.5 Was the outcome determined without knowledge of predictor information?	Yes	The outcome was death, which is objective.
3.6 Was the time interval between predictor assessment and outcome determination appropriate?	Yes	The time interval, from ART initiation till the end of follow-up (12 years in total) was long enough to observe the death outcome.
**Overall risk of bias of Domain 3**	**High risk of bias**	
**Domain 4: Analysis**		
4.1 Were there a reasonable number of participants with the outcome?	No	The number of events per variable = 105 death/35 = 3, which is too small.
4.2 Were continuous and categorical predictors handled appropriately?	Probably no	Continuous predictors (CD4^+^ and hemoglobin) were not examined for nonlinearity, but generally, these two variables are right skewed and should be log-transformed before entering the model.
4.3 Were all enrolled participants included in the analysis?	No	Among the 3584 patients in the control group, only 600 could be matched and included in analyses, whereas the remaining could not be successfully matched were excluded.
4.4 Were participants with missing data handled appropriately?	Probably no	Although multiple imputation was used, there was no explicit mention of the specific method used to analyze imputed data.
4.5 Was selection of predictors based on univariable analysis avoided?	No	Selection was entirely based on *p* values in univariate Cox analyses and ROC analyses.
4.6 Were complexities in the data (e.g., censoring, competing risks, sampling of controls) accounted for appropriately?	No	1. Censored data were not mentioned and might not be handled properly. 2. Non-AIDS-related death was not accounted as a competing risk of AIDS-related death. 3. Propensity-score matching approach was misused.
4.7 Were relevant model performance measures evaluated appropriately?	Probably yes	Discrimination was assessed by the concordance index, and calibration curve was used to assess calibration.
4.8 Were model overfitting and optimism in model performance accounted for?	No	Internal validation consists only of a single random split sample of participant data and did not include all model development procedures including any variable selection.
4.9 Do predictors and their assigned weights in the final model correspond to the results from multivariable analysis?	No	The final model was based only on a selection of predictors from the reported multivariable regression analysis without refitting the smaller model.
**Overall risk of bias of Domain 4**	**High risk of bias**	

In the Wenzhou model development study, the nested case–control design was applied at a 1:4 ratio to determine the study population, in which one case (dead PLWHA) was matched with four controls by age and gender [13]. The inappropriate use of the nested case–control design and misuse of propensity score matching in prediction model development study lead to unfavorable answers to SQ1.1, SQ4.3, and SQ4.6 in PROBAST. Specifically, with the nested case–control design, the authors artificially fixed the event rate (HIV-related mortality) at 20% (150/750) by selecting 600 controls out of 3583 living PLWHA, which would lead to a much higher event rate than in real-world PLWHA population. In fact, in another study of 13812 PLWHA in Zhejiang, the province where Wenzhou is part of, the HIV-related mortality was merely around 5.4% [36]. As a result, the prognostic model developed based on this selective cohort without proper adjustment is highly likely to overestimate the probability of death (i.e., underestimate the survival probability).

Additionally, propensity score matching is not a reasonable approach for selecting controls for prediction model development studies. When developing a new model, the ultimate goal is to include all predictors that could contribute to predicting the outcome. This is contradictory with propensity score matching, as the variables used for matching would be balanced in case and control groups, thus can no longer serve as predictors. The empirical impact of using age and gender as matching variables rather than (potential) predictors in developing the Wenzhou model is shown in the external validation section.

There are also some issues in defining the outcome to be predicted, which leads to high risk of bias in SQ3.1 and SQ4.6 in PROBAST. The authors chose HIV-related death as the endpoint, but did not explicitly mention how death from other causes (the competing risk event) was dealt with in the analysis [13]. Since the model was developed with a Cox model, it is most likely that the standard Cox model was used by censoring the death from other causes. However, this approach would substantially overestimate the probability (absolute risk) of the event, leading to poor calibration accuracy and wrong prediction in clinical practice [37, 38]. Because clinical prediction models are used for decision-making in the real world, but not a virtual world where the competing risk is absent [37], the model developed with simply censoring death from other causes would provide a prediction of HIV/AIDS-specific survival probability, which is misleading, irrelevant, and of course biased. In this case, the Fine and Gray model accounting for competing risks would be more suitable [37].

In the model development, authors randomly split the cohort into a training set and a validation set at a ratio of 7:3 [13]. This approach cannot be seen as an independent external validation. In fact, this was only a weak and inefficient form of internal validation [4], as 70% of all available data was used for model development. This approach reduced the sample size, which was already small, from 750 to 525 to develop the model, resulting in a very low (105 death/35 variables = 3) event-per-variable (SQ3.1), and optimism cannot be adjusted appropriately either [4]. A low event-per-variable would lead to model overfitting and overestimating the model performance and cannot ensure the desirable model performance in an external validation [4].

### Model performance

#### Distribution of prognostic index

**Fig. 2:**
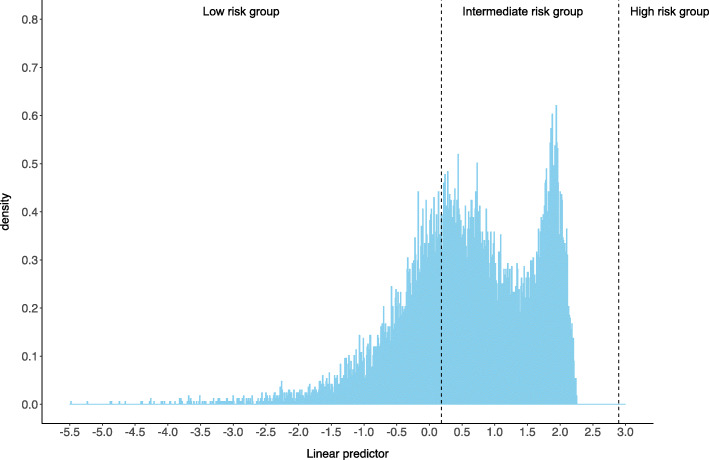
Distribution of the linear predictor

Figure 2 shows the distribution of PI in the validation cohort. Based on the risk groups proposed in the Wenzhou study, 5518 (32.93%) patients were in the low-risk group, 11240 (60.07%) patients were in the intermediate group, and no patient was classified as high risk.

#### Discrimination performance

**Fig. 3:**
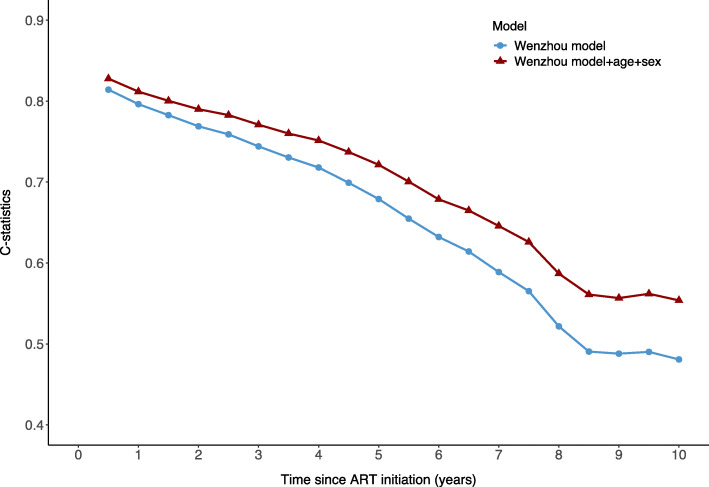
Time-dependent C-statistics comparing the Wenzhou model and the extended model

Harrell’s overall C-statistics is 0.76 (95% CI 0.74–0.77) in the validation cohort, which is much lower than the apparent C-statistics (0.93) and in random split validation (0.95) reported in the Wenzhou model development study [13]. The time-dependent C-statistics decreased from 0.81 to 0.74 from 6 months to 3 years after ART initiation and continued decreasing to 0.48 at 10 years (Fig. 3).

#### Calibration accuracy

**Fig. 4:**
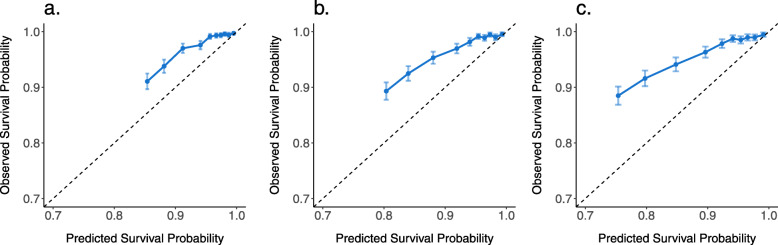
Calibration curves at 1 year (**a**), 2 years (**b**), and 3 years (**c**)

Figure 4 shows the calibration curves at 1, 2, and 3 years after ART initiation. At all three time points, the Wenzhou model consistently underestimated the survival probability (i.e., overestimated the mortality rate) in the validation cohort. On average, the Wenzhou model underestimated the survival probability by 3.13%, 4.34%, and 5.82% at 1, 2, and 3 years after ART initiation, respectively, and the lower the predicted survival the higher level of underestimation, which can be up to 6.23%, 10.02%, and 14.82% at 1, 2, and 3 years after ART initiation, respectively. This confirmed our concern of overestimation of the event rate in the “Methodology quality assessment” section.

### Incremental prediction value of age and gender

After adding age and gender to the original model, Harrell’s overall C-statistics increased from 0.76 to 0.78 (95% CI 0.76–0.79), and the time-dependent C-statistics also increased for all time points (Fig. 3).

The same results were also observed in the sensitivity analysis of complete cases (supplement figure 2). In the two sensitivity analyses that respectively assume all the missing values for HIV viral load were < 200 copies/mL (supplement figure 3) and that had the same distribution as reported in the Wenzhou study (supplement figure 4), the predicted probability of death was lower and the calibration became even worse. The discrimination performance was consistent with that in the main analysis.

## Discussion

In this critical appraisal and external validation, we evaluated the Wenzhou model from both its risk of bias and model performance. Based on the framework of PROBAST, in which the highest methodology standard was applied for critical appraisal, the model was rated as high risk of bias in three out of the four domains. In the external validation in a large population-based cohort, the model performance was poor in both discrimination and calibration.

According to the PROBAST, the Wenzhou model was prone to high risk of bias in the selection of study participants, definition of outcome, and methods for statistical analysis [19]. This largely contributes to the poor model performance in the external validation. Age and gender are two important risk factors for the survival of PLWHA, which has been consistently identified in previous prospective studies [33–35] as well as prognostic model development studies [6, 8, 9, 39]. However, age and gender were used as matching variables and therefore precluded as candidate predictors in developing the Wenzhou model [13], which undoubtedly crippled the discriminative ability of the model. This could be confirmed by our results that both the overall and time-dependent discriminative ability (C-statistics) increased after adding age and gender to the original Wenzhou model. Obviously, the approach of matching variables is at odds with the principle of prediction model studies.

The Wenzhou model was developed with a nested case–control study design, however, adjustment of the baseline risk or recalibration of the probability prediction had not been performed to obtain the correct probability estimate, so the mortality risk was overestimated. This could be supported by our results of assessing the calibration accuracy of the Wenzhou model which reveal a severe and consistent overestimation of risk. The inappropriate use of propensity score matching and random split validation substantially reduced the sample size for model development, which further lead to decreased model performance in the external validation.

A reliable and validated prognostic model would be a powerful tool for assisting physicians in the decision-making process. However, in spite of seemingly rigorous methodology and excellent model performance in the development and internal validation of the Wenzhou model, findings from our external validation study show that directly applying this model to clinical practice would engender negative consequences. We found that the Wenzhou model tends to overestimate the mortality risk of PLWHA up to 15%. For those patients with advanced HIV diseases who already have unfavorable prognosis, if the Wenzhou model was used to counsel patients about prognosis, for example, the estimated prognosis would be even worse and thereby cause pessimism and deflated confidence in treatment among those patients, and some of them might even give up treatment altogether. On the other hand, intensive care and management would be disproportionately given to those with mild diseases due to the overestimated risk, bringing about tremendous waste in healthcare resources.

Prediction model study has different methodological considerations compared with other types of clinical or epidemiological studies. Indiscriminately applying experiences gained from other fields in the study design and statistical analysis to the development of clinical prediction models is not only likely to generate biased and misleading models of no clinical usefulness, but also might set a fallacious example for other researchers new to developing prediction model to imitate. Instead, researchers should carefully refer to guidelines specifically developed for clinical prediction models, including the reporting standard TRIPOD [4, 15] and the methodological standard PROBAST [19, 20]. Given that the analysis in model development is relatively more complicated compared with that in other clinical and epidemiological studies, the involvement of statisticians and methodologists in prediction model studies are necessary.

Additionally, a downward trend in time-dependent C-statistics over follow-up time was observed in the external validation. This indicates that a prediction model based on only baseline information may lose its prediction ability for long-term outcome. Incorporating predictor values collected during follow-up in the prediction model may improve the model performance, and such model can be developed using dynamic prediction approaches including joint modeling and landmarking analysis [40].

Our study has several limitations. First, our dataset has high percentage of missing values for HIV viral load (85.7%). The presence of missing values is inevitable for clinical data, especially for our dataset with large sample size (16758). The reasons for missing values for HIV viral load are largely due to limited medical resources in the hospital as well as limited financial means of patients, especially for data collected in earlier years. To fulfill the requirement of missing at random, we included a total of 26 auxiliary variables into the imputation model, to make sure missingness is conditional on the observed data. We are confident about our findings because we handled missing values properly by multiple imputation for 50 times, and results were consistent in our complete analysis excluding all missing values and two additional sensitivity analyses with different assumptions in missing values. In comparison, the Wenzhou study did not report any missing value for HIV viral load. This is perhaps because of the small selected sample (525), or the authors simply deleted eligible patients with missing values, an approach that would incur serious bias [15]. Second, because of the limited information on causes of death, we could not reliably distinguish HIV-related and non-HIV-related deaths, so the endpoint of the external validation study was all-cause mortality, which should be higher than HIV-related mortality as being predicted by the Wenzhou model. Nevertheless, the model predicted event probability was still noticeably higher than that observed in our external population-based cohort, even if patients in the external validation data were more clinically advanced and had higher HIV viral load at ART initiation compared with patients in the Wenzhou study. If we would use HIV-related death as the outcome, the overestimation of HIV-related mortality would be more pronounced than that of all-cause mortality presented in this paper. Third, data used for external validation in this study were derived from one hospital, though the Guangzhou Eighth People’s Hospital is one of the largest designated hospitals for HIV/AIDS treatment in China and has been treating around one third of PLHIV in the Guangdong province. Lastly, both the Wenzhou study and this study were based on PLHIV in China, with limited generalizability. But our study has a broader implication in terms of providing a comprehensive overview of how model development studies in general could be improved. The combined qualitative and quantitative approach used in this study could also be applied in other external validation studies in the future.

Since the publication of PROBAST in early 2019, it has been widely used in many systematic reviews of clinical prediction models [41–43], but it may take a long time for a prediction model to be included and assessed in a systematic review. Externally validating a newly developed model in a separate dataset could be a practical alternative. However, most external validation studies merely focus on model performance while ignoring the inherent methodological quality of the studies developing that model. This could be misleading in some instances. It is entirely possible that, for example, a prediction model with desirable performance in external validation was developed in a study of poor methodological quality. In order to have a comprehensive appraisal of a prediction model, combining the assessment of methodological quality and risk of bias with external validation is necessary. To the best of our knowledge, this study is the first attempt to incorporate critical appraisal as part of external validation, and it can serve as an example of the new standard of external validation which contains both qualitative and quantitative analyses. Although PROBAST was originally designed as a risk of bias assessment tool, we found it also provided a structured way in evaluating the methodological quality of a prediction model. The applicability of PROBAST in evaluating methodological quality of prediction models will be assessed when such evaluation is performed more frequently. However, the assessment largely depends on the reported information from the original study, whereas incomplete reporting and unclear description may mislead the evaluators. Hence, the compliance with TRIPOD reporting guideline [15] is highly desired for model developers.

## Conclusion

In summary, the Wenzhou model is rated as high risk of bias in model development, with sub-optimal model performance in our external validation. The validity and extended utility of the Wenzhou model are also hard to confirm. Future prediction model development and validation studies should carefully refer to and follow well-established methodological standards and guidelines specifically developed for the prediction model.

## Supplementary Information

**Additional file 1:.** Supplementary tables and figures and R code used in the external validation

## Data Availability

The dataset used for the current study is not publicly available due to restrictions from the China Center for Disease Control and Prevention.

## Abbreviations

PLWHA: People living with HIV/AIDS
ART: Antiretroviral therapy
PROBAST: Prediction Model Risk of Bias Assessment Tool
CDC: Center for Disease Control and Prevention
WHO: World Health Organization
TRIPOD: Transparent Reporting of a multivariable prediction model for Individual Prognosis or Diagnosis statement
SQ: Signaling questions
IQR: Interquartile range
CI: Confidence interval
