# Supplementary material for: Critical appraisal and external validation of a prognostic model for survival of people living with HIV/AIDS who underwent antiretroviral therapy
Source: Diagn Progn Res. 2020 Nov 25;4:19. doi: 10.1186/s41512-020-00088-x (PMC7687783; doi:10.1186/s41512-020-00088-x)
Supplement: Supplementary file 1 — Additional file 1:. Supplementary tables and figures and R code used in the external validation [file 41512_2020_88_MOESM1_ESM.docx]

[Supplementary Table 1. Transparent Reporting of a multivariable prediction model for Individual Prognosis or Diagnosis statement Checklist 2](#_Toc52464600)

[Supplementary Table 2. Ratings of the Wenzhou study by two independent researchers before discussion 3](#_Toc52464601)

[Supplementary Table 3. Descriptive analysis of predictors in prognostic model 4](#_Toc52464602)

[Supplement Figure 1. Kaplan-Meier survival curves comparing complete cases and cases with missing predictors 5](#_Toc52464603)

[Supplement Figure 2. Sensitivity analysis for complete cases (n=2,374) 6](#_Toc52464604)

[Supplementary Figure 2.1. Distribution of linear predictor 6](#_Toc52464605)

[Supplement Figure 2.2. Time-dependent C-index comparing Wenzhou model and extended model 7](#_Toc52464606)

[Supplement Figure 2.3. Calibration curves at 1 year (a), 2 year (b), and 3 year (c) 8](#_Toc52464607)

[Supplement Figure 3. Sensitivity analysis of assuming all the missing values for HIV viral load were <200 copies/mL 9](#_Toc52464608)

[Supplementary Figure 3.1. Distribution of linear predictor 9](#_Toc52464609)

[Supplement Figure 3.2. Time-dependent C-index comparing Wenzhou model and extended model 10](#_Toc52464610)

[Supplement Figure 3.3. Calibration curves at 1 year (a), 2 year (b), and 3 year (c) 11](#_Toc52464611)

[Supplement Figure 4. Sensitivity analysis of assuming missing values for HIV viral load had the same distribution as reported in the Wenzhou study. 12](#_Toc52464612)

[Supplementary Figure 4.1. Distribution of linear predictor 12](#_Toc52464613)

[Supplement Figure 4.2. Time-dependent C-index comparing Wenzhou model and extended model 13](#_Toc52464614)

[Supplement Figure 4.3. Calibration curves at 1 year (a), 2 year (b), and 3 year (c) 14](#_Toc52464615)

[R code used in the external validation 15](#_Toc52464616)

# Supplementary Table 1. Transparent Reporting of a multivariable prediction model for Individual Prognosis or Diagnosis statement Checklist

| **Section/Topic** | **Item** | **Checklist Item** | **Page** |
| --- | --- | --- | --- |
| **Title and abstract** | | | |
| Title | 1 | Identify the study as developing and/or validating a multivariable prediction model, the target population, and the outcome to be predicted. | 1 |
| Abstract | 2 | Provide a summary of objectives, study design, setting, participants, sample size, predictors, outcome, statistical analysis, results, and conclusions. | 4 |
| **Introduction** | | | |
| Background and objectives | 3a | Explain the medical context (including whether diagnostic or prognostic) and rationale for developing or validating the multivariable prediction model, including references to existing models. | 7-8 |
|  | 3b | Specify the objectives, including whether the study describes the development or validation of the model or both. | 8 |
| **Methods** | | | |
| Source of data | 4a | Describe the study design or source of data (e.g., randomized trial, cohort, or registry data), separately for the development and validation data sets, if applicable. | 9-10 |
|  | 4b | Specify the key study dates, including start of accrual; end of accrual; and, if applicable, end of follow-up. | 10 |
| Participants | 5a | Specify key elements of the study setting (e.g., primary care, secondary care, general population) including number and location of centres. | 10 |
|  | 5b | Describe eligibility criteria for participants. | 10 |
|  | 5c | Give details of treatments received, if relevant. | Not applicable |
| Outcome | 6a | Clearly define the outcome that is predicted by the prediction model, including how and when assessed. | 10-11 |
|  | 6b | Report any actions to blind assessment of the outcome to be predicted. | Not applicable |
| Predictors | 7a | Clearly define all predictors used in developing or validating the multivariable prediction model, including how and when they were measured. | 10 |
|  | 7b | Report any actions to blind assessment of predictors for the outcome and other predictors. | Not applicable |
| Sample size | 8 | Explain how the study size was arrived at. | 11 |
| Missing data | 9 | Describe how missing data were handled (e.g., complete-case analysis, single imputation, multiple imputation) with details of any imputation method. | 12-13 |
| Statistical analysis methods | 10c | For validation, describe how the predictions were calculated. | 13-14 |
|  | 10d | Specify all measures used to assess model performance and, if relevant, to compare multiple models. | 11-12 |
|  | 10e | Describe any model updating (e.g., recalibration) arising from the validation, if done. | Not applicable |
| Risk groups | 11 | Provide details on how risk groups were created, if done. | 14 |
| Development vs. validation | 12 | For validation, identify any differences from the development data in setting, eligibility criteria, outcome, and predictors. | 10-11 |
| **Results** | | | |
| Participants | 13a | Describe the flow of participants through the study, including the number of participants with and without the outcome and, if applicable, a summary of the follow-up time. A diagram may be helpful. | 17, figure1 |
|  | 13b | Describe the characteristics of the participants (basic demographics, clinical features, available predictors), including the number of participants with missing data for predictors and outcome. | 17-18 |
|  | 13c | For validation, show a comparison with the development data of the distribution of important variables (demographics, predictors and outcome). | 17-18, table1 |
| Model performance | 16 | Report performance measures (with CIs) for the prediction model. | 18-19 |
| Model-updating | 17 | If done, report the results from any model updating (i.e., model specification, model performance). |  |
| **Discussion** | | | |
| Limitations | 18 | Discuss any limitations of the study (such as nonrepresentative sample, few events per predictor, missing data). | 22-23 |
| Interpretation | 19a | For validation, discuss the results with reference to performance in the development data, and any other validation data. | 19-20 |
|  | 19b | Give an overall interpretation of the results, considering objectives, limitations, results from similar studies, and other relevant evidence. | 23 |
| Implications | 20 | Discuss the potential clinical use of the model and implications for future research. | 21-22 |
| **Other information** | | | |
| Supplementary information | 21 | Provide information about the availability of supplementary resources, such as study protocol, Web calculator, and data sets. | supplementary |
| Funding | 22 | Give the source of funding and the role of the funders for the present study. | 14 |

# Supplementary Table 2. Ratings of the Wenzhou study by two independent researchers before discussion

|  | Two raters | |
| --- | --- | --- |
|  | **JW** | **TY** |
| DOMAIN 1: Participants |  |  |
| 1.1 Were appropriate data sources used, e.g. cohort, RCT or nested case-control study data? | No/Probably no | No/Probably no |
| 1.2 Were all inclusions and exclusions of participants appropriate? | Yes/Probably yes | No/Probably no |
| Overall ROB Domain 1 | **High risk of bias** | **High risk of bias** |
| DOMAIN 2: Predictors |  |  |
| 2.1 Were predictors defined and assessed in a similar way for all participants? | Yes/Probably yes | Yes/Probably yes |
| 2.2 Were predictor assessments made without knowledge of outcome data? | Yes/Probably yes | Yes/Probably yes |
| 2.3 Are all predictors available at the time the model is intended to be used? | Yes/Probably yes | Yes/Probably yes |
| Overall ROB Domain 2 | **Low risk of bias** | **Low risk of bias** |
| DOMAIN 3: Outcome |  |  |
| 3.1 Was the outcome determined appropriately? | Yes/Probably yes | No information |
| 3.2 Was a pre-specified or standard outcome definition used? | Yes/Probably yes | No information |
| 3.3 Were predictors excluded from the outcome definition? | Yes/Probably yes | Yes/Probably yes |
| 3.4 Was the outcome defined and determined in a similar way for all participants? | Yes/Probably yes | No information |
| 3.5 Was the outcome determined without knowledge of predictor information? | Yes/Probably yes | Yes/Probably yes |
| 3.6 Was the time interval between predictor assessment and outcome determination appropriate? | Yes/Probably yes | Yes/Probably yes |
| Overall ROB Domain 3 | **Low risk of bias** | **Unclear risk of bias** |
| DOMAIN 4: Analysis |  |  |
| 4.1 Were there a reasonable number of participants with the outcome? | No/Probably no | No/Probably no |
| 4.2 Were continuous and categorical predictors handled appropriately? | No/Probably no | Yes/Probably yes |
| 4.3 Were all enrolled participants included in the analysis? | No/Probably no | No/Probably no |
| 4.4 Were participants with missing data handled appropriately? | No/Probably no | Yes/Probably yes |
| 4.5 Was selection of predictors based on univariable analysis avoided? | No/Probably no | No/Probably no |
| 4.6 Were complexities in the data (e.g. censoring, competing risks, sampling of controls) accounted for appropriately? | No/Probably no | No/Probably no |
| 4.7 Were relevant model performance measures evaluated appropriately? | Yes/Probably yes | Yes/Probably yes |
| 4.8 Were model overfitting and optimism in model performance accounted for? | No/Probably no | No/Probably no |
| 4.9 Do predictors and their assigned weights in the final model correspond to the results from multivariable analysis? | No/Probably no | No/Probably no |
| Overall ROB Domain 4 | **High risk of bias** | **High risk of bias** |

# Supplementary Table 3. Descriptive analysis of predictors in prognostic model

|  | **Before multiple imputation** | **After multiple imputation** |
| --- | --- | --- |
| Viral load, copies/mL | 64700.00 (17300.00, 222000.00) | 159494.00 (65028.00, 380887.00) |
| <200 | 39 (1.63) | 39 (0.23) |
| 200-1000 | 57 (2.38) | 57 (0.34) |
| ≥1000 | 2301 (95.99) | 16662 (99.43) |
| CD4 cell count, cells/μL | 209.00 (72.00, 319.00) | 208.00 (72.00, 318.00) |
| Haemoglobin, g/L | 137.00 (116.00, 150.00) | 137.00 (116.00, 150.00) |

Categorical variables are presented as n (%), and continuous variables are presented as median (interquartile range).


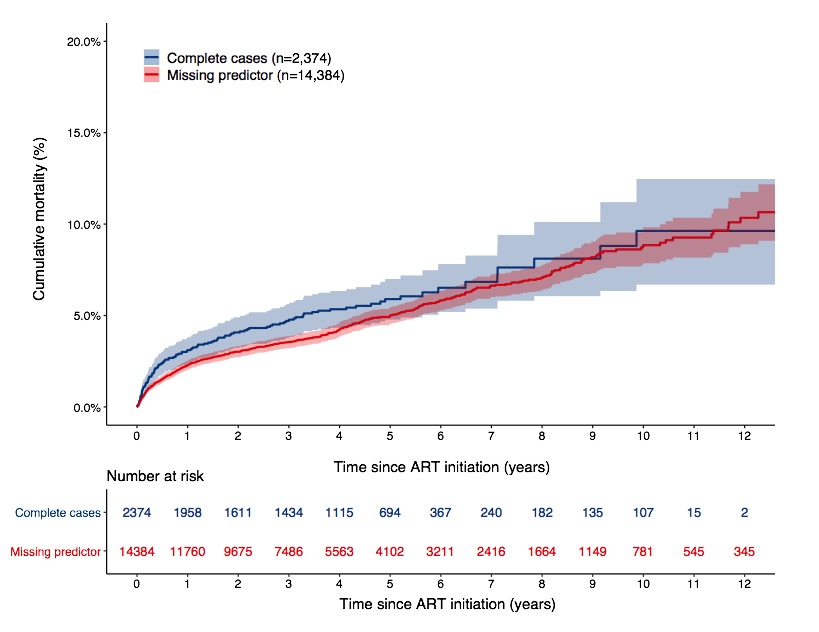


# Supplement Figure 1. Kaplan-Meier survival curves comparing complete cases and cases with missing predictors

# Supplement Figure 2. Sensitivity analysis for complete cases (n=2,374)

## Supplementary Figure 2.1. Distribution of linear predictor

## Supplement Figure 2.2. Time-dependent C-index comparing Wenzhou model and extended model


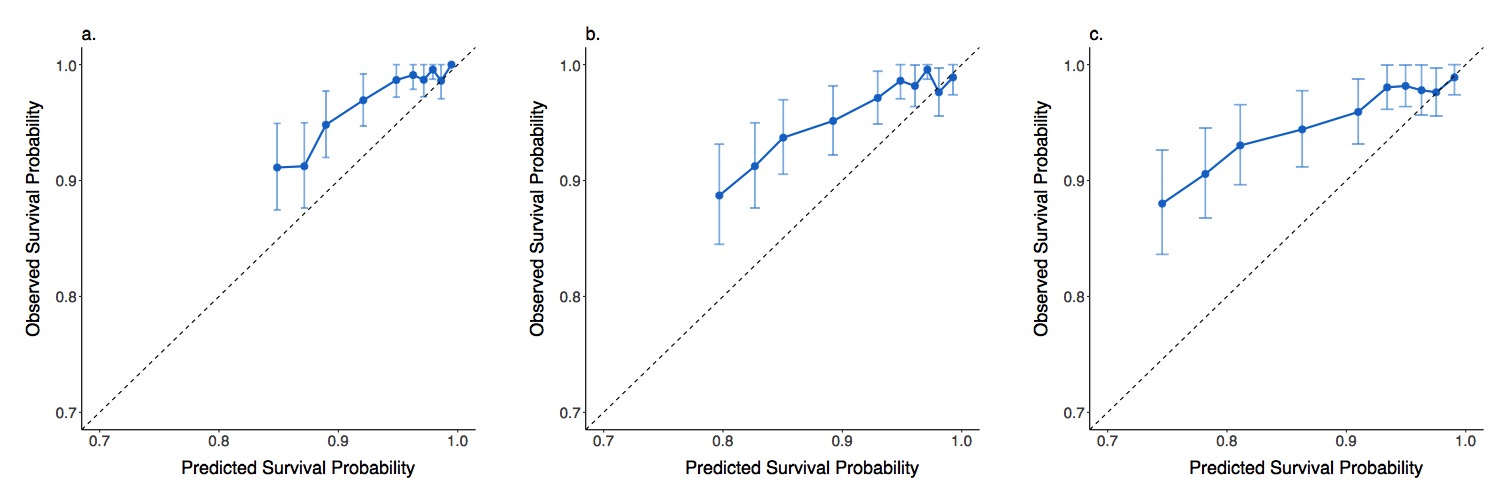


## Supplement Figure 2.3. Calibration curves at 1 year (a), 2 year (b), and 3 year (c)

# Supplement Figure 3. Sensitivity analysis of assuming all the missing values for HIV viral load were <200 copies/mL

## Supplementary Figure 3.1. Distribution of linear predictor

## Supplement Figure 3.2. Time-dependent C-index comparing Wenzhou model and extended model

## Supplement Figure 3.3. Calibration curves at 1 year (a), 2 year (b), and 3 year (c)

# Supplement Figure 4. Sensitivity analysis of assuming missing values for HIV viral load had the same distribution as reported in the Wenzhou study.

## Supplementary Figure 4.1. Distribution of linear predictor

## Supplement Figure 4.2. Time-dependent C-index comparing Wenzhou model and extended model

## Supplement Figure 4.3. Calibration curves at 1 year (a), 2 year (b), and 3 year (c)

# R code used in the external validation

# load packages

library(mice)

library(rms)

library(compareC)

library(riskRegression)

####### Main analysis: based on 50 imputed datasets #######

###### 0. Prepare data for impuation

baseimput<-subset(baselineend, select =

c("id", "gender","HBV","Btuberculosis","BWHO","infectionroute",

"Particicat", "maritalstatuscat", "logviral", "age", "weight","height",

"logCD4", "logCD8", "logWBC", "logPlatelet", "loghaemoglobin",

'logSCR', 'logTG', 'logTch', 'logGLU', 'logAST', 'logALT', 'logT.BIL',

'residence', 'ARTinitiacat', 'ARTreg', "time","death"))

# calculate cumulative hazard###########

HT1 <- summary(survival::survfit(Surv(time,death)~1,data=baseimput))

baseimput$haz_os <- approx(c(0,HT1$time),-log(c(1,HT1$surv)),xout=baseimput$time,method="constant",f=0,rule=2)$y

##### 1. Imputation of missing values #####

# see all the default settings for imputation

impu_default <- mice(baseimput, maxit = 0)

summary(impu_default)

# check predictor structure

pred <- quickpred(baseimput, exclude = c("id","time"))

pred

# check imputation method

meth <- impu_default$meth

# set number of imputation

K <- 50

# impute the missing values with mice

imputation_50 <- mice(baseimput, maxit = 25, m = K, seed = 1234, pred = pred, meth = meth, print = TRUE)

##### 2. Analyze the imputed data #####

sample_size <- nrow(baseimput)

lp_nomogram_impu <- seq(1:sample_size)

S1_nomogram <- seq(1:sample_size)

S2_nomogram <- seq(1:sample_size)

S3_nomogram <- seq(1:sample_size)

viral <- seq(1:sample_size)

CD4 <- seq(1:sample_size)

HG <- seq(1:sample_size)

# extract imputed data and calculate the predicted probability

n_impu <- K

data_impu <- vector(n_impu,mode="list")

for (i in 1:n_impu) {

data_impu[[i]] <- mice::complete(imputation_50, i)

data_impu[[i]]$death<-as.numeric(data_impu[[i]]$death)-1

# transform log back

data_impu[[i]]$viral.load.con <- exp(data_impu[[i]]$logviral)

data_impu[[i]]$BCD4 <- exp(data_impu[[i]]$logCD4)

data_impu[[i]]$haemoglobin <- exp(data_impu[[i]]$loghaemoglobin)

# extract viral load, CD4 and haemoglobin

viral <- data.frame(viral,data_impu[[i]]$viral.load.con)

CD4 <- data.frame(CD4,data_impu[[i]]$BCD4)

HG <-data.frame(HG, data_impu[[i]]$haemoglobin)

# categorize viral load

data_impu[[i]]$viral.load <- ifelse(data_impu[[i]]$viral.load.con<200,1,ifelse(data_impu[[i]]$viral.load.con>=1000,3,2))

# calculate score for viral.load

data_impu[[i]]$score_viral.load <- ifelse(data_impu[[i]]$viral.load==2,1.019669556,ifelse(data_impu[[i]]$viral.load==3,2.608969326,0))

# calculate linear predictor

data_impu[[i]]$lp_nomogram <- data_impu[[i]]$score_viral.load -0.005580907*data_impu[[i]]$BCD4 -0.005368102*data_impu[[i]]$haemoglobin

lp_nomogram_impu <- data.frame(lp_nomogram_impu,data_impu[[i]]$lp_nomogram)##

# calculate predicted survival probability

## based on formula extracted from nomogram

data_impu[[i]]$S1_nomogram <- 0.980222074^exp(data_impu[[i]]$lp_nomogram)

data_impu[[i]]$S2_nomogram <- 0.972736744^exp(data_impu[[i]]$lp_nomogram)

data_impu[[i]]$S3_nomogram <- 0.964896148^exp(data_impu[[i]]$lp_nomogram)

# add age and sex to linear predictor

## age code (<40=1, 40-59=2,>=60=3) & ###score###code gender(Men=1, Female=2)##score

data_impu[[i]]<-within.data.frame(data_impu[[i]], {

age.code<-ifelse(age<40, 1,

ifelse(age>=60,3,2))

score_age<-ifelse(age.code==2,log(1.269),

ifelse(age.code==3,log(1.798),0))

sex.code<-ifelse(gender=='Men',1,2)

score_sex<-ifelse(sex.code==2, log(0.7), 0)

})

data_impu[[i]]$lp_nomogram_age_sex <- data_impu[[i]]$lp_nomogram + data_impu[[i]]$score_age + data_impu[[i]]$score_sex

}

# analyze the imputed data

calibration_slope <- 0

calibration_slope_var <- 0

c_harrel <- 0

c_harrel_var <- 0

c_harrel_age_sex <- 0

c_harrel_age_sex_var <- 0

c_harrel_diff <- 0

c_harrel_diff_var <- 0

c_time <- 0

c_time_age_sex <- 0

c_time_combine <- seq(1:20)/2

c_time_age_sex_combine <- seq(1:20)/2

IDI_combine <- seq(1:20)/2

survival_predicted_1_combine <- seq(1:10)

survival_predicted_2_combine <- seq(1:10)

survival_predicted_3_combine <- seq(1:10)

survival_observed_1_combine <- seq(1:10)

survival_observed_2_combine <- seq(1:10)

survival_observed_3_combine <- seq(1:10)

survival_observed_1_var_combine <- seq(1:10)

survival_observed_2_var_combine <- seq(1:10)

survival_observed_3_var_combine <- seq(1:10)

for (i in 1:n_impu) {

# calibration slopel (R package rms)

validation <- cph(Surv(time, death)~lp_nomogram, data=data_impu[[i]],x=TRUE,y=TRUE)

validation_age_sex<- cph(Surv(time, death)~lp_nomogram_age_sex, data=data_impu[[i]],x=TRUE,y=TRUE)

calibration_slope[i] <- validation$coefficients

calibration_slope_var[i] <- validation$var

## Harrell's C-statistics (R package compareC)

c_harrel[i] <- 1- estC(data_impu[[i]]$time, data_impu[[i]]$death,data_impu[[i]]$lp_nomogram)

c_harrel_age_sex[i] <- 1- estC(data_impu[[i]]$time, data_impu[[i]]$death,data_impu[[i]]$lp_nomogram_age_sex)

c_harrel_diff[i] <- compareC(data_impu[[i]]$time, data_impu[[i]]$death, data_impu[[i]]$lp_nomogram, data_impu[[i]]$lp_nomogram_age_sex)$est.diff_c

c_harrel_diff_var[i] <- vardiffC(data_impu[[i]]$time, data_impu[[i]]$death, data_impu[[i]]$lp_nomogram, data_impu[[i]]$lp_nomogram_age_sex)$est.vardiff_c

c_harrel_var[i] <- vardiffC(data_impu[[i]]$time, data_impu[[i]]$death, data_impu[[i]]$lp_nomogram, data_impu[[i]]$lp_nomogram_age_sex)$est.varCxy

c_harrel_age_sex_var[i] <- vardiffC(data_impu[[i]]$time, data_impu[[i]]$death, data_impu[[i]]$lp_nomogram, data_impu[[i]]$lp_nomogram_age_sex)$est.varCxz

## Time dependent C-statistics (R package riskRegression)

c_time <- 0

c_time_age_sex <- 0

for (j in 1:20){

c_time[j] <- Score(list(validation),Surv(time,death)~1,data=data_impu[[i]],times=(0.5*j), plots="cal",metrics=c("AUC"))$AUC$score$AUC

c_time_age_sex[j] <- Score(list(validation_age_sex),Surv(time,death)~1,data=data_impu[[i]],times=(0.5*j), plots="cal",metrics=c("AUC"))$AUC$score$AUC

}

c_time_combine <- data.frame(c_time_combine,c_time)

c_time_age_sex_combine <- data.frame(c_time_age_sex_combine,c_time_age_sex)

## Calibration plot

data_impu[[i]]$group10<-cut(data_impu[[i]]$lp_nomogram, quantile(data_impu[[i]]$lp_nomogram, seq(0,1,0.1)), right=FALSE, labels=c(1:10))

data_impu[[i]]$group10[data_impu[[i]]$lp_nomogram==max(data_impu[[i]]$lp_nomogram)] <- 10

survival_predicted_1 <- 0

survival_predicted_2 <- 0

survival_predicted_3 <- 0

# 1-year predicted survival

survival_predicted_1 <- aggregate(data_impu[[i]]$S1_nomogram, list(data_impu[[i]]$group10), mean)

survival_predicted_1_combine <- data.frame(survival_predicted_1_combine,survival_predicted_1$x)

# 2-year predicted survival

survival_predicted_2 <- aggregate(data_impu[[i]]$S2_nomogram, list(data_impu[[i]]$group10), mean)

survival_predicted_2_combine <- data.frame(survival_predicted_2_combine,survival_predicted_2$x)

# 3-year predicted survival

survival_predicted_3 <- aggregate(data_impu[[i]]$S3_nomogram, list(data_impu[[i]]$group10), mean)

survival_predicted_3_combine <- data.frame(survival_predicted_3_combine,survival_predicted_3$x)

# observed survival

survival_observed_1 <- 0

survival_observed_1_var <- 0

survival_observed_2 <- 0

survival_observed_2_var <- 0

survival_observed_3 <- 0

survival_observed_3_var <- 0

for (j in 1:10) {

data_temp <- subset(data_impu[[i]],data_impu[[i]]$group10==j)

fit_calibration <- survfit(Surv(time,death) ~ 1, data=data_temp)

survival_observed_1[j] <- min(fit_calibration$surv[fit_calibration$time <= 1])

survival_observed_1_var[j] <- (tail(fit_calibration$std.err[fit_calibration$time <= 1],1))^2

survival_observed_2[j] <- min(fit_calibration$surv[fit_calibration$time <= 2])

survival_observed_2_var[j] <- (tail(fit_calibration$std.err[fit_calibration$time <= 2],1))^2

survival_observed_3[j] <- min(fit_calibration$surv[fit_calibration$time <= 3])

survival_observed_3_var[j] <- (tail(fit_calibration$std.err[fit_calibration$time <= 3],1))^2

}

survival_observed_1_combine <- data.frame(survival_observed_1_combine,survival_observed_1)

survival_observed_2_combine <- data.frame(survival_observed_2_combine,survival_observed_2)

survival_observed_3_combine <- data.frame(survival_observed_3_combine,survival_observed_3)

survival_observed_1_var_combine <- data.frame(survival_observed_1_var_combine,survival_observed_1_var)

survival_observed_2_var_combine <- data.frame(survival_observed_2_var_combine,survival_observed_2_var)

survival_observed_3_var_combine <- data.frame(survival_observed_3_var_combine,survival_observed_3_var)

}

# combine results from 50 imputation

## summary viral, cd4, and haemoglobin

viral_final<-rowMeans(viral[,-1])

viralold<-subset(baselineend, !is.na(baselineend$viralload), select = 'viralload')

viralold$var<-'complete'

viralold<-data.frame(seq(1:2397), viralold)

virald<-data.frame(seq(1:sample_size), viral_final)

virald$seq.1.sample_size.<-NULL

virald$cat<-NULL

colnames(virald)[1]<-'viralload'

viral<-rbind(viralold,virald)

viral$var<-as.factor(viral$var)

kruskal.test(viral, var)

virald$cat<-as.factor(ifelse(virald$viral_final<200, "<200",

ifelse(virald$viral_final>=200 & virald$viral_final<1000,"200-1000", "≥1000")))

summary(virald$cat)

CD4_final<-rowMeans(CD4[,-1])

summary(CD4_final)

HG_final<-rowMeans(HG[,-1])

IQR(viral_final)

summary(baselineend$viralload)

summary(HG_final)

##### line graph of linear predictor

lp_nomogram_final<-rowMeans(lp_nomogram_impu[,-1])

lp_nomogram_impu<-data.frame(seq(1:sample_size), lp_nomogram_final)

lp_nomogram_impu$risksc<-108.3333333 + 19.90914787*lp_nomogram_impu$lp_nomogram_final

summary(lp_nomogram_impu$lp_nomogram_final)

summary(lp_nomogram_impu$risksc)

lp_nomogram_impu$riskg<-as.factor(ifelse(lp_nomogram_impu$lp_nomogram_final<=0.184169968 ,"Low", "Intermediate"

))

summary(lp_nomogram_impu$riskg)

p1<-ggplot(lp_nomogram_impu,

aes( x=lp_nomogram_final, color=riskg, y=..density..), break.x.by=0.1,) +

geom_histogram(binwidth=0.02, position="identity")+

scale_x_continuous(breaks = seq(-5.5,2.5,0.5), limits = c(-5.5,2.5), name='Linear predictor')+

scale_y_continuous(breaks=seq(0,1, 0.1), limits = c(0,0.8))+

theme(axis.text = element_text(size = 15), axis.title=element_text(size=15),

panel.background = element_rect(fill = "white"),

axis.title.y = element_text(margin = margin(t = 0, r = 0.5, b = 0, l = 0, "cm")),

axis.title.x= element_text(margin = margin(t = 0.5, r = 0, b = 0, l = 0, "cm") ))+

geom_vline(aes(xintercept=0.184169968),linetype="dashed")+

scale_fill_manual(values = c("Low" = "skyblue2",

"Medium" = "firebrick"))

# calibration slope

calibration_slope_final <- mean(calibration_slope)

range(calibration_slope)

calibration_slope_var_final <- mean(calibration_slope_var) + (1+1/n_impu)*var(calibration_slope_var)# = 0.002750295

c(calibration_slope_final-qnorm(0.975)*calibration_slope_var_final^0.5,calibration_slope_final+qnorm(0.975)*calibration_slope_var_final^0.5) # 95% CI

# C-statistics

# Harrell's C

# Wenzhou model

c_harrel_final <- mean(c_harrel)

range(c_harrel)

c_harrel_var_final <- mean(c_harrel_var) + (1+1/n_impu)*var(c_harrel_var)

c(c_harrel_final-qnorm(0.975)*c_harrel_var_final^0.5, c_harrel_final+qnorm(0.975)*c_harrel_var_final^0.5) # 95% CI

# Wenzhou model + age + sex

c_harrel_age_sex_final <- mean(c_harrel_age_sex)

range(c_harrel_age_sex)

c_harrel_age_sex_var_final <- mean(c_harrel_age_sex_var) + (1+1/n_impu)*var(c_harrel_age_sex_var)

c(c_harrel_age_sex_final-qnorm(0.975)*c_harrel_age_sex_var_final^0.5, c_harrel_age_sex_final+qnorm(0.975)*c_harrel_age_sex_var_final^0.5) # 95% CI

# comparison

c_harrel_diff_final <- mean(c_harrel_diff)

range(c_harrel_diff)

c_harrel_diff_var_final <- mean(c_harrel_diff_var) + (1+1/n_impu)*var(c_harrel_diff_var)

c(c_harrel_diff_final-qnorm(0.975)*c_harrel_diff_var_final^0.5,c_harrel_diff_final+qnorm(0.975)*c_harrel_diff_var_final^0.5) # 95% CI

## time dependent C-statistics

# Wenzhou model

c_time_final <- rowMeans(c_time_combine[,-1])

c_time_combime <- data.frame(year,c_time_final)

# Wenzhou model+age+sex

c_time_age_sex_final <- rowMeans(c_time_age_sex_combine[,-1])

# then make a line chart with time as x, c_time and c_time_age_sex as y

year <- seq(1:20)/2

c_time_combime <- data.frame(year,c_time_final,c_time_age_sex_final)

c_time_combimer<- reshape2::melt(c_time_combime, id.var='year')

save(c_time_combimer, file='dependentCline.RData')

summary(c_time_combimer$value)

f1<-ggplot(c_time_combimer, aes(x=year, y=value, col=variable)) + geom_line(size=1)+geom_point(aes(shape=variable), size=3)+

labs(y = 'C-statistics',

x = "Time since ART initiation (years)")+

scale_color_manual(values=c("steelblue3", "darkred"),

name ="Model",

breaks=c("c_time_final","c_time_age_sex_final"),

labels=c("Wenzhou model", "Wenzhou model+age+sex"))+

scale_y_continuous(breaks = seq(0.4,0.9, 0.1), limits = c(0.4,0.9) )+

scale_x_continuous(breaks=seq(0,10, 1), limits = c(0,10))+

scale_shape_discrete(name ="Model",

breaks=c("c_time_final","c_time_age_sex_final"),

labels=c("Wenzhou model", "Wenzhou model+age+sex"))+

theme(legend.position=c(0.8,0.9),

legend.text = element_text(size=14),

legend.title = element_text(size=14),

legend.key=element_blank(),

legend.background=element_blank(),

axis.text = element_text(size = 15), axis.title=element_text(size=15),

panel.background = element_rect(fill = "white"),

axis.line = element_line(color='black'),

axis.title.y = element_text(margin = margin(t = 0, r = 0.5, b = 0, l = 0, "cm")),

axis.title.x= element_text(margin = margin(t = 0.5, r = 0, b = 0, l = 0, "cm")),

plot.margin = margin(1, 1, 1, 1, "cm"))

# calibration plot

# 1-year

survival_predicted_1_final <- exp(rowMeans(log(survival_predicted_1_combine[,-1])))

survival_observed_1_final <- exp(rowMeans(log(survival_observed_1_combine[,-1])))

survival_observed_1_var_final <- rowMeans(survival_observed_1_var_combine[,-1]) + (1+1/n_impu)*apply(survival_observed_1_var_combine[,-1], MARGIN=1, FUN=var, na.rm=TRUE)

survival_lower1_final<-exp(log(survival_observed_1_final) - qnorm(0.975)*survival_observed_1_var_final^0.5)

survival_upper1_final<-exp(log(survival_observed_1_final) + qnorm(0.975)*survival_observed_1_var_final^0.5)

survival_comparison1 <- data.frame(survival_predicted_1_final, survival_observed_1_final,

survival_lower1_final,survival_upper1_final)

survival_comparison1$survival_upper1_final<-ifelse(survival_comparison1$survival_upper1_final>1, 1,survival_comparison1$survival_upper1_final)

survival_comparison1$underestimate<-(survival_comparison1$survival_observed_1_final-survival_comparison1$survival_predicted_1_final)/survival_comparison1$survival_observed_1_final

c1<-ggplot(data=survival_comparison1, aes(x=survival_predicted_1_final, y=survival_observed_1_final)) +

geom_line(size=1, colour="dodgerblue3")+

geom_errorbar(data=survival_comparison1, mapping=aes(x=survival_predicted_1_final, ymin=survival_lower1_final,

ymax=survival_upper1_final),

colour="dodgerblue3", size=1,alpha=0.5, linetype=1)+

geom_point(size=3, colour="dodgerblue3")+

xlim(0.7,1)+

ylim(0.7,1)+

geom_abline(intercept = 0, slope = 1,lty=2)+

labs(title="A. Calibration curve at 1 year",x="Predicted Survival Probability", y = "Observed Survival Probability")+

theme( axis.text = element_text(size = 15), axis.title=element_text(size=15),

panel.background = element_rect(fill = "white"),

plot.title = element_text(size=15),

axis.title.y = element_text(margin = margin(t = 0, r = 0.5, b = 0, l = 0, "cm")),

axis.title.x= element_text(margin = margin(t = 0.5, r = 0, b = 0, l = 0, "cm")),

plot.margin = margin(1, 1, 1, 1, "cm"),

axis.line = element_line(color='black'),

)

(mean(survival_comparison1$survival_observed_1_final)-mean(survival_comparison1$survival_predicted_1_final))*100

(mean(survival_comparison2$survival_observed_2_final)-mean(survival_comparison2$survival_predicted_2_final))*100

(mean(survival_comparison3$survival_observed_3_final)-mean(survival_comparison3$survival_predicted_3_final))*100

# 2-year

survival_predicted_2_final <- exp(rowMeans(log(survival_predicted_2_combine[,-1])))

survival_observed_2_final <- exp(rowMeans(log(survival_observed_2_combine[,-1])))

survival_observed_2_var_final <- rowMeans(survival_observed_2_var_combine[,-1]) + (1+1/n_impu)*apply(survival_observed_2_var_combine[,-1], MARGIN=1, FUN=var, na.rm=TRUE)

survival_lower2_final<-exp(log(survival_observed_2_final) - qnorm(0.975)*survival_observed_2_var_final^0.5)

survival_upper2_final<-exp(log(survival_observed_2_final) + qnorm(0.975)*survival_observed_2_var_final^0.5)

survival_comparison2 <- data.frame(survival_predicted_2_final, survival_observed_2_final,

survival_lower2_final,survival_upper2_final)

survival_comparison2$survival_upper2_final<-ifelse(survival_comparison2$survival_upper2_final>1, 1,survival_comparison2$survival_upper2_final)

survival_comparison2$underestimate<-(survival_comparison2$survival_observed_2_final-survival_comparison2$survival_predicted_2_final)/survival_comparison2$survival_observed_2_final

c2<-ggplot(data=survival_comparison2, aes(x=survival_predicted_2_final, y=survival_observed_2_final)) +

geom_line(size=1, colour="dodgerblue3")+

geom_errorbar(data=survival_comparison2, mapping=aes(x=survival_predicted_2_final, ymin=survival_lower2_final,

ymax=survival_upper2_final),

colour="dodgerblue3", size=1,alpha=0.5, linetype=1)+

geom_point(size=3, colour="dodgerblue3")+

xlim(0.7,1)+

ylim(0.7,1)+

geom_abline(intercept = 0, slope = 1,lty=2)+

labs(title="B. Calibration curve at 2 year",x="Predicted Survival Probability", y = "Observed Survival Probability")+

theme( axis.text = element_text(size = 15), axis.title=element_text(size=15),

panel.background = element_rect(fill = "white"),

plot.title = element_text(size=15),

axis.title.y = element_text(margin = margin(t = 0, r = 0.5, b = 0, l = 0, "cm")),

axis.title.x= element_text(margin = margin(t = 0.5, r = 0, b = 0, l = 0, "cm")),

plot.margin = margin(1, 1, 1, 1, "cm"),

axis.line = element_line(color='black'),

)

# 3-year

survival_predicted_3_final <- exp(rowMeans(log(survival_predicted_3_combine[,-1])))

survival_observed_3_final <- exp(rowMeans(log(survival_observed_3_combine[,-1])))

survival_observed_3_var_final <- rowMeans(survival_observed_3_var_combine[,-1]) + (1+1/n_impu)*apply(survival_observed_3_var_combine[,-1], MARGIN=1, FUN=var, na.rm=TRUE)

survival_lower3_final<-exp(log(survival_observed_3_final) - qnorm(0.975)*survival_observed_3_var_final^0.5)

survival_upper3_final<-exp(log(survival_observed_3_final) + qnorm(0.975)*survival_observed_3_var_final^0.5)

survival_comparison3 <- data.frame(survival_predicted_3_final, survival_observed_3_final,

survival_lower3_final,survival_upper3_final)

survival_comparison3$survival_upper3_final<-ifelse(survival_comparison3$survival_upper3_final>1, 1,survival_comparison3$survival_upper3_final)

survival_comparison3$underestimate<-(survival_comparison3$survival_observed_3_final-survival_comparison3$survival_predicted_3_final)/survival_comparison3$survival_observed_3_final

c3<-ggplot(data=survival_comparison3, aes(x=survival_predicted_3_final, y=survival_observed_3_final)) +

geom_line(size=1, colour="dodgerblue3")+

geom_errorbar(data=survival_comparison3, mapping=aes(x=survival_predicted_3_final, ymin=survival_lower3_final,

ymax=survival_upper3_final),

colour="dodgerblue3", size=1,alpha=0.5, linetype=1)+

geom_point(size=3, colour="dodgerblue3")+

xlim(0.7,1)+

ylim(0.7,1)+

geom_abline(intercept = 0, slope = 1,lty=2)+

labs(title="C. Calibration curve at 3 year",x="Predicted Survival Probability", y = "Observed Survival Probability")+

theme( axis.text = element_text(size = 15), axis.title=element_text(size=15),

panel.background = element_rect(fill = "white"),

plot.title = element_text(size=15),

axis.title.y = element_text(margin = margin(t = 0, r = 0.5, b = 0, l = 0, "cm")),

axis.title.x= element_text(margin = margin(t = 0.5, r = 0, b = 0, l = 0, "cm")),

plot.margin = margin(1, 1, 1, 1, "cm"),

axis.line = element_line(color='black'),

)

ggarrange(c1, c2, c3, ncol = 3, nrow = 1)

####### Sensitivity analysis: based on complete cases #######

# coding of predictors

## viral.load (<200=1, 200-1000=2,>=1000=3)

basecomplete<-within.data.frame(basecomplete, {

viral.load<-ifelse(viralloadcat=="<200", 1,

ifelse(viralloadcat=="200-1000", 2,3))

})

# calculate linear predictor

attach(basecomplete)

## based on formula extracted from nomogram###

basecomplete$score_viral.load <- ifelse(viral.load==2,1.019669556,ifelse(viral.load==3,2.608969326,0))

attach(basecomplete)

basecomplete$lp_nomogram <- score_viral.load-0.005580907*BCD4 -0.005368102*haemoglobin

summary(basecomplete$lp_nomogram )

# add age and sex in linear predictor

## age code (<40=1, 40-59=2,>=60=3) & ###score###code gender(Men=1, Female=2)##score

basecomplete<-within.data.frame(basecomplete, {

age.code<-ifelse(age<40, 1,

ifelse(age>=60,3,2))

score_age<-ifelse(age.code==2,log(1.269),

ifelse(age.code==3,log(1.798),0))

sex.code<-ifelse(gender=='Men',1,2)

score_sex<-ifelse(sex.code==2, log(0.7), 0)

})

basecomplete$lp_nomogram_age_sex <- basecomplete$lp_nomogram + basecomplete$score_age + basecomplete$score_sex

# calculate predicted survival probability

attach(basecomplete)

## based on formula extracted from nomogram

basecomplete$S1_nomogram <- 0.980222074^exp(lp_nomogram)

basecomplete$S2_nomogram <- 0.972736744^exp(lp_nomogram)

basecomplete$S3_nomogram <- 0.964896148^exp(lp_nomogram)

# plot distribution of linear predictor

summary(lp_nomogram)

ggplot(basecomplete, aes(x=lp_nomogram, y=..density..), break.x.by=0.1,) +

geom_histogram(binwidth=0.01, color="skyblue", fill="skyblue")+

scale_x_continuous(breaks = seq(-6,2.3,0.5), name='Linear predictor')+

scale_y_continuous(breaks=seq(0,1, 0.1), limits = c(0,0.8))

# caculate model performance measures

## calibration slopel

validation <- cph(Surv(time, death)~lp_nomogram, data=basecomplete,x=TRUE,y=TRUE)

validation$coefficients

# Harrell's C-statistics

## for complete data

c_harrel <- 1-estC(basecomplete$time, basecomplete$death,basecomplete$lp_nomogram)

c_harrel_age_sex <- 1- estC(basecomplete$time, basecomplete$death,basecomplete$lp_nomogram_age_sex)

# compare model w/o age and sex

compareC(basecomplete$time, basecomplete$death, basecomplete$lp_nomogram, basecomplete$lp_nomogram_age_sex)

# Time dependent C-statistics

c_time <- 0

c_time_age_sex <- 0

for (i in 1:20){

c_time[i] <- Score(list(validation),Surv(time,death)~1,data=basecomplete,times=(0.5*i), plots="cal",metrics=c("AUC"))$AUC$score$AUC

c_time_age_sex[i] <- Score(list(validation_age_sex),Surv(time,death)~1,data=basecomplete,times=(0.5*i), plots="cal",metrics=c("AUC"))$AUC$score$AUC

}

c_time

c_time_age_sex

year <- seq(1:20)/2

c_time_combime <- data.frame(year,c_time,c_time_age_sex)

c_time_combimer<- reshape2::melt(c_time_combime, id.var='year')

# then make a line chart with time as x, c_time and c_time_age_sex as y

f1<-ggplot(c_time_combimer, aes(x=year, y=value, col=variable)) + geom_line()+geom_point(aes(shape=variable), size=2)+

labs(y = 'C-statistics',

x = "Year",

title = 'Time dependent C-statistics')+

scale_color_manual(values=c("steelblue3", "darkred"),

name ="Group",

breaks=c("c_time_age_sex", "c_time"),

labels=c("Wenzhou model+age+sex","Wenzhou model"))+

scale_y_continuous(breaks = seq(0.5,0.9, 0.1), limits = c(0.5,0.9) )+

scale_x_continuous(breaks=seq(0,10, 1), limits = c(0,10))+

scale_shape_discrete(name ="Group",

breaks=c("c_time_age_sex", "c_time"),

labels=c("Wenzhou model+age+sex","Wenzhou model"))

## Calibration plot

basecomplete$group10<-cut(basecomplete$lp_nomogram, quantile(basecomplete$lp_nomogram, seq(0,1,0.1)), right=FALSE, labels=c(1:10))

basecomplete$group10[basecomplete$lp_nomogram==max(basecomplete$lp_nomogram)] <- 10

table(basecomplete$group10)

# at 1 year

survival_predicted <- aggregate(basecomplete$S1_nomogram, list(basecomplete$group10), mean)

survival_observed <-0

survival_lower <-0

survival_upper <-0

for (i in 1:10) {

data_temp <- subset(basecomplete,basecomplete$group10==i)

fit_calibration <- survfit(Surv(time,death) ~ 1, data=data_temp)

survival_observed[i] <- min(fit_calibration$surv[fit_calibration$time <= 1])

survival_lower[i] <- min(fit_calibration$lower[fit_calibration$time <= 1])

survival_upper[i] <- min(fit_calibration$upper[fit_calibration$time <= 1])

}

survival_comparison <- data.frame(survival_predicted, survival_observed,survival_lower,survival_upper)

c1<-ggplot(data=survival_comparison, aes(x=x, y=survival_observed)) +

geom_line()+

geom_point()+

geom_errorbar(aes(ymin=survival_lower, ymax=survival_upper), width=.2,

position=position_dodge(0.05))+

xlim(0.85,1)+

ylim(0.85,1)+

geom_abline(intercept = 0, slope = 1,lty=2)+

labs(title="A. Calibration curve at 1 year",x="Predicted Survival Probability", y = "Observed Survival Probability")

# at 2-year

survival_predicted2 <- aggregate(basecomplete$S2_nomogram, list(basecomplete$group10), mean)

survival_observed2 <-0

survival_lower2 <-0

survival_upper2 <-0

for (i in 1:10) {

data_temp <- subset(basecomplete,basecomplete$group10==i)

fit_calibration <- survfit(Surv(time,death) ~ 1, data=data_temp)

survival_observed2[i] <- min(fit_calibration$surv[fit_calibration$time <= 2])

survival_lower2[i] <- min(fit_calibration$lower[fit_calibration$time <= 2])

survival_upper2[i] <- min(fit_calibration$upper[fit_calibration$time <= 2])

}

survival_comparison2 <- data.frame(survival_predicted2, survival_observed2,survival_lower2,survival_upper2)

c2<-ggplot(data=survival_comparison2, aes(x=x, y=survival_observed2)) +

geom_line()+

geom_point()+

geom_errorbar(aes(ymin=survival_lower2, ymax=survival_upper2), width=.2,

position=position_dodge(0.05))+

xlim(0.85,1)+

ylim(0.85,1)+

geom_abline(intercept = 0, slope = 1,lty=2)+

labs(title="B. Calibration curve at 2 year",x="Predicted Survival Probability", y = "Observed Survival Probability")

# at 3 year

survival_predicted3 <- aggregate(basecomplete$S3_nomogram, list(basecomplete$group10), mean)

survival_observed3 <-0

survival_lower3 <-0

survival_upper3 <-0

for (i in 1:10) {

data_temp <- subset(basecomplete,basecomplete$group10==i)

fit_calibration <- survfit(Surv(time,death) ~ 1, data=data_temp)

survival_observed3[i] <- min(fit_calibration$surv[fit_calibration$time <= 3])

survival_lower3[i] <- min(fit_calibration$lower[fit_calibration$time <= 3])

survival_upper3[i] <- min(fit_calibration$upper[fit_calibration$time <= 3])

}

survival_comparison3 <- data.frame(survival_predicted3, survival_observed3,survival_lower3,survival_upper3)

c3<-ggplot(data=survival_comparison3, aes(x=x, y=survival_observed3)) +

geom_line()+

geom_point()+

geom_errorbar(aes(ymin=survival_lower3, ymax=survival_upper3), width=.2,

position=position_dodge(0.05))+

xlim(0.85,1)+

ylim(0.85,1)+

geom_abline(intercept = 0, slope = 1,lty=2)+

labs(title="C. Calibration curve at 3 year",x="Predicted Survival Probability", y = "Observed Survival Probability")

ggarrange(c1, c2, c3, ncol = 3, nrow = 1)

####### Sensitivity analysis: assuming all the missing values for HIV viral load were <200 copies/mL #######

################## HIV viral missing<200 ################################

baselineend$viralload[is.na(baselineend$viralload)]<-198

newviral<-subset(baselineend, select = c('id', 'viralload'))

colnames(newviral)[2]<-'viral.load.con'

##### ## Analyze the imputed data #####

sample_size <- nrow(baselineend)

lp_nomogram_impu <- seq(1:sample_size)

S1_nomogram <- seq(1:sample_size)

S2_nomogram <- seq(1:sample_size)

S3_nomogram <- seq(1:sample_size)

viral <- seq(1:sample_size)

CD4 <- seq(1:sample_size)

HG <- seq(1:sample_size)

# extract imputed data and calculate the predicted probability

n_impu <- 50

data_impu <- vector(n_impu,mode="list")

for (i in 1:n_impu) {

data_impu[[i]] <- mice::complete(imputation_50, i)

data_impu[[i]]$death<-as.numeric(data_impu[[i]]$death)-1

# merge new viral

data_impu[[i]]<-merge(data_impu[[i]], newviral, by='id')

# transform log back

data_impu[[i]]$BCD4 <- exp(data_impu[[i]]$logCD4)

data_impu[[i]]$haemoglobin <- exp(data_impu[[i]]$loghaemoglobin)

# extract viral load, CD4 and haemoglobin

viral <- data.frame(viral,data_impu[[i]]$viral.load.con)

CD4 <- data.frame(CD4,data_impu[[i]]$BCD4)

HG <-data.frame(HG, data_impu[[i]]$haemoglobin)

# categorize viral load

data_impu[[i]]$viral.load <- ifelse(data_impu[[i]]$viral.load.con<200,1,ifelse(data_impu[[i]]$viral.load.con>=1000,3,2))

# calculate score for viral.load

data_impu[[i]]$score_viral.load <- ifelse(data_impu[[i]]$viral.load==2,1.019669556,ifelse(data_impu[[i]]$viral.load==3,2.608969326,0))

# calculate linear predictor

data_impu[[i]]$lp_nomogram <- data_impu[[i]]$score_viral.load -0.005580907*data_impu[[i]]$BCD4 -0.005368102*data_impu[[i]]$haemoglobin

lp_nomogram_impu <- data.frame(lp_nomogram_impu,data_impu[[i]]$lp_nomogram)##

# calculate predicted survival probability

## based on formula extracted from nomogram

data_impu[[i]]$S1_nomogram <- 0.980222074^exp(data_impu[[i]]$lp_nomogram)

data_impu[[i]]$S2_nomogram <- 0.972736744^exp(data_impu[[i]]$lp_nomogram)

data_impu[[i]]$S3_nomogram <- 0.964896148^exp(data_impu[[i]]$lp_nomogram)

# add age and sex to linear predictor

## age code (<40=1, 40-59=2,>=60=3) & ###score###code gender(Men=1, Female=2)##score

data_impu[[i]]<-within.data.frame(data_impu[[i]], {

age.code<-ifelse(age<40, 1,

ifelse(age>=60,3,2))

score_age<-ifelse(age.code==2,log(1.269),

ifelse(age.code==3,log(1.798),0))

sex.code<-ifelse(gender=='Men',1,2)

score_sex<-ifelse(sex.code==2, log(0.7), 0)

})

data_impu[[i]]$lp_nomogram_age_sex <- data_impu[[i]]$lp_nomogram + data_impu[[i]]$score_age + data_impu[[i]]$score_sex

}

summary(data_impu[[18]])

# analyze the imputed data

calibration_slope <- 0

calibration_slope_var <- 0

c_harrel <- 0

c_harrel_var <- 0

c_harrel_age_sex <- 0

c_harrel_age_sex_var <- 0

c_harrel_diff <- 0

c_harrel_diff_var <- 0

c_time <- 0

c_time_age_sex <- 0

c_time_combine <- seq(1:20)/2

c_time_age_sex_combine <- seq(1:20)/2

IDI_combine <- seq(1:20)/2

survival_predicted_1_combine <- seq(1:10)

survival_predicted_2_combine <- seq(1:10)

survival_predicted_3_combine <- seq(1:10)

survival_observed_1_combine <- seq(1:10)

survival_observed_2_combine <- seq(1:10)

survival_observed_3_combine <- seq(1:10)

survival_observed_1_var_combine <- seq(1:10)

survival_observed_2_var_combine <- seq(1:10)

survival_observed_3_var_combine <- seq(1:10)

for (i in 1:n_impu) {

## Time dependent C-statistics (R package riskRegression)

c_time <- 0

c_time_age_sex <- 0

for (j in 1:20){

c_time[j] <- Score(list(validation),Surv(time,death)~1,data=data_impu[[i]],times=(0.5*j), plots="cal",metrics=c("AUC"))$AUC$score$AUC

c_time_age_sex[j] <- Score(list(validation_age_sex),Surv(time,death)~1,data=data_impu[[i]],times=(0.5*j), plots="cal",metrics=c("AUC"))$AUC$score$AUC

}

c_time_combine <- data.frame(c_time_combine,c_time)

c_time_age_sex_combine <- data.frame(c_time_age_sex_combine,c_time_age_sex)

## Calibration plot

data_impu[[i]]$group10<-cut(data_impu[[i]]$lp_nomogram, quantile(data_impu[[i]]$lp_nomogram, seq(0,1,0.1)), right=FALSE, labels=c(1:10))

data_impu[[i]]$group10[data_impu[[i]]$lp_nomogram==max(data_impu[[i]]$lp_nomogram)] <- 10

survival_predicted_1 <- 0

survival_predicted_2 <- 0

survival_predicted_3 <- 0

# 1-year predicted survival

survival_predicted_1 <- aggregate(data_impu[[i]]$S1_nomogram, list(data_impu[[i]]$group10), mean)

survival_predicted_1_combine <- data.frame(survival_predicted_1_combine,survival_predicted_1$x)

# 2-year predicted survival

survival_predicted_2 <- aggregate(data_impu[[i]]$S2_nomogram, list(data_impu[[i]]$group10), mean)

survival_predicted_2_combine <- data.frame(survival_predicted_2_combine,survival_predicted_2$x)

# 3-year predicted survival

survival_predicted_3 <- aggregate(data_impu[[i]]$S3_nomogram, list(data_impu[[i]]$group10), mean)

survival_predicted_3_combine <- data.frame(survival_predicted_3_combine,survival_predicted_3$x)

# observed survival

survival_observed_1 <- 0

survival_observed_1_var <- 0

survival_observed_2 <- 0

survival_observed_2_var <- 0

survival_observed_3 <- 0

survival_observed_3_var <- 0

for (j in 1:10) {

data_temp <- subset(data_impu[[i]],data_impu[[i]]$group10==j)

fit_calibration <- survfit(Surv(time,death) ~ 1, data=data_temp)

survival_observed_1[j] <- min(fit_calibration$surv[fit_calibration$time <= 1])

survival_observed_1_var[j] <- (tail(fit_calibration$std.err[fit_calibration$time <= 1],1))^2

survival_observed_2[j] <- min(fit_calibration$surv[fit_calibration$time <= 2])

survival_observed_2_var[j] <- (tail(fit_calibration$std.err[fit_calibration$time <= 2],1))^2

survival_observed_3[j] <- min(fit_calibration$surv[fit_calibration$time <= 3])

survival_observed_3_var[j] <- (tail(fit_calibration$std.err[fit_calibration$time <= 3],1))^2

}

survival_observed_1_combine <- data.frame(survival_observed_1_combine,survival_observed_1)

survival_observed_2_combine <- data.frame(survival_observed_2_combine,survival_observed_2)

survival_observed_3_combine <- data.frame(survival_observed_3_combine,survival_observed_3)

survival_observed_1_var_combine <- data.frame(survival_observed_1_var_combine,survival_observed_1_var)

survival_observed_2_var_combine <- data.frame(survival_observed_2_var_combine,survival_observed_2_var)

survival_observed_3_var_combine <- data.frame(survival_observed_3_var_combine,survival_observed_3_var)

}

# combine results from 50 imputation

##### line graph of linear predictor

lp_nomogram_final<-rowMeans(lp_nomogram_impu[,-1])

lp_nomogram_impu<-data.frame(seq(1:sample_size), lp_nomogram_final)

lp_nomogram_impu$risksc<-108.3333333 + 19.90914787*lp_nomogram_impu$lp_nomogram_final

summary(lp_nomogram_impu$lp_nomogram_final)

summary(lp_nomogram_impu$risksc)

lp_nomogram_impu$riskg<-as.factor(ifelse(lp_nomogram_impu$lp_nomogram_final<=0.184169968 ,"Low", "Intermediate"

))

summary(lp_nomogram_impu$riskg)

ggplot(lp_nomogram_impu, aes(x=lp_nomogram_final, y=..density..), break.x.by=0.1,) +

geom_histogram(binwidth=0.01, color="skyblue", fill="skyblue")+

scale_x_continuous(breaks = seq(-5.5,3,0.5), limits = c(-5.5,3), name='Linear predictor')+

scale_y_continuous(breaks=seq(0,1, 0.1), limits = c(0,0.8))+

theme(axis.text = element_text(size = 15), axis.title=element_text(size=15),

panel.background = element_rect(fill = "white"),

axis.line = element_line(color='black'),

axis.title.y = element_text(margin = margin(t = 0, r = 0.5, b = 0, l = 0, "cm")),

axis.title.x= element_text(margin = margin(t = 0.5, r = 0, b = 0, l = 0, "cm") ))+

geom_vline(aes(xintercept=0.184169968),linetype="dashed")+

geom_vline(aes(xintercept=2.896490986),linetype="dashed")

## time dependent C-statistics

# Wenzhou model

c_time_final <- rowMeans(c_time_combine[,-1])

c_time_combime <- data.frame(year,c_time_final)

# Wenzhou model+age+sex

c_time_age_sex_final <- rowMeans(c_time_age_sex_combine[,-1])

# then make a line chart with time as x, c_time and c_time_age_sex as y

year <- seq(1:20)/2

c_time_combime <- data.frame(year,c_time_final,c_time_age_sex_final)

c_time_combimer<- reshape2::melt(c_time_combime, id.var='year')

save(c_time_combimer, file='dependentCline.RData')

summary(c_time_combimer$value)

f1<-ggplot(c_time_combimer, aes(x=year, y=value, col=variable)) + geom_line(size=1)+geom_point(aes(shape=variable), size=3)+

labs(y = 'C-statistics',

x = "Time since ART initiation (years)")+

scale_color_manual(values=c("steelblue3", "darkred"),

name ="Model",

breaks=c("c_time_final","c_time_age_sex_final"),

labels=c("Wenzhou model", "Wenzhou model+age+sex"))+

scale_y_continuous(breaks = seq(0.4,0.9, 0.1), limits = c(0.4,0.9) )+

scale_x_continuous(breaks=seq(0,10, 1), limits = c(0,10))+

scale_shape_discrete(name ="Model",

breaks=c("c_time_final","c_time_age_sex_final"),

labels=c("Wenzhou model", "Wenzhou model+age+sex"))+

theme(legend.position=c(0.8,0.9),

legend.text = element_text(size=14),

legend.title = element_text(size=14),

legend.key=element_blank(),

legend.background=element_blank(),

axis.text = element_text(size = 15), axis.title=element_text(size=15),

panel.background = element_rect(fill = "white"),

axis.line = element_line(color='black'),

axis.title.y = element_text(margin = margin(t = 0, r = 0.5, b = 0, l = 0, "cm")),

axis.title.x= element_text(margin = margin(t = 0.5, r = 0, b = 0, l = 0, "cm")),

plot.margin = margin(1, 1, 1, 1, "cm"))

# calibration plot

# 1-year

survival_predicted_1_final <- exp(rowMeans(log(survival_predicted_1_combine[,-1])))

survival_observed_1_final <- exp(rowMeans(log(survival_observed_1_combine[,-1])))

survival_observed_1_var_final <- rowMeans(survival_observed_1_var_combine[,-1]) + (1+1/n_impu)*apply(survival_observed_1_var_combine[,-1], MARGIN=1, FUN=var, na.rm=TRUE)

survival_lower1_final<-exp(log(survival_observed_1_final) - qnorm(0.975)*survival_observed_1_var_final^0.5)

survival_upper1_final<-exp(log(survival_observed_1_final) + qnorm(0.975)*survival_observed_1_var_final^0.5)

survival_comparison1 <- data.frame(survival_predicted_1_final, survival_observed_1_final,

survival_lower1_final,survival_upper1_final)

survival_comparison1$survival_upper1_final<-ifelse(survival_comparison1$survival_upper1_final>1, 1,survival_comparison1$survival_upper1_final)

survival_comparison1$underestimate<-(survival_comparison1$survival_observed_1_final-survival_comparison1$survival_predicted_1_final)/survival_comparison1$survival_observed_1_final

c1<-ggplot(data=survival_comparison1, aes(x=survival_predicted_1_final, y=survival_observed_1_final)) +

geom_line(size=1, colour="dodgerblue3")+

geom_errorbar(data=survival_comparison1, mapping=aes(x=survival_predicted_1_final, ymin=survival_lower1_final,

ymax=survival_upper1_final),

colour="dodgerblue3", size=1,alpha=0.5, linetype=1)+

geom_point(size=3, colour="dodgerblue3")+

xlim(0.7,1)+

ylim(0.7,1)+

geom_abline(intercept = 0, slope = 1,lty=2)+

labs(title="A. Calibration curve at 1 year",x="Predicted Survival Probability", y = "Observed Survival Probability")+

theme( axis.text = element_text(size = 15), axis.title=element_text(size=15),

panel.background = element_rect(fill = "white"),

plot.title = element_text(size=15),

axis.title.y = element_text(margin = margin(t = 0, r = 0.5, b = 0, l = 0, "cm")),

axis.title.x= element_text(margin = margin(t = 0.5, r = 0, b = 0, l = 0, "cm")),

plot.margin = margin(1, 1, 1, 1, "cm"),

axis.line = element_line(color='black'),

)

# 2-year

survival_predicted_2_final <- exp(rowMeans(log(survival_predicted_2_combine[,-1])))

survival_observed_2_final <- exp(rowMeans(log(survival_observed_2_combine[,-1])))

survival_observed_2_var_final <- rowMeans(survival_observed_2_var_combine[,-1]) + (1+1/n_impu)*apply(survival_observed_2_var_combine[,-1], MARGIN=1, FUN=var, na.rm=TRUE)

survival_lower2_final<-exp(log(survival_observed_2_final) - qnorm(0.975)*survival_observed_2_var_final^0.5)

survival_upper2_final<-exp(log(survival_observed_2_final) + qnorm(0.975)*survival_observed_2_var_final^0.5)

survival_comparison2 <- data.frame(survival_predicted_2_final, survival_observed_2_final,

survival_lower2_final,survival_upper2_final)

survival_comparison2$survival_upper2_final<-ifelse(survival_comparison2$survival_upper2_final>1, 1,survival_comparison2$survival_upper2_final)

survival_comparison2$underestimate<-(survival_comparison2$survival_observed_2_final-survival_comparison2$survival_predicted_2_final)/survival_comparison2$survival_observed_2_final

c2<-ggplot(data=survival_comparison2, aes(x=survival_predicted_2_final, y=survival_observed_2_final)) +

geom_line(size=1, colour="dodgerblue3")+

geom_errorbar(data=survival_comparison2, mapping=aes(x=survival_predicted_2_final, ymin=survival_lower2_final,

ymax=survival_upper2_final),

colour="dodgerblue3", size=1,alpha=0.5, linetype=1)+

geom_point(size=3, colour="dodgerblue3")+

xlim(0.7,1)+

ylim(0.7,1)+

geom_abline(intercept = 0, slope = 1,lty=2)+

labs(title="B. Calibration curve at 2 year",x="Predicted Survival Probability", y = "Observed Survival Probability")+

theme( axis.text = element_text(size = 15), axis.title=element_text(size=15),

panel.background = element_rect(fill = "white"),

plot.title = element_text(size=15),

axis.title.y = element_text(margin = margin(t = 0, r = 0.5, b = 0, l = 0, "cm")),

axis.title.x= element_text(margin = margin(t = 0.5, r = 0, b = 0, l = 0, "cm")),

plot.margin = margin(1, 1, 1, 1, "cm"),

axis.line = element_line(color='black'),

)

# 3-year

survival_predicted_3_final <- exp(rowMeans(log(survival_predicted_3_combine[,-1])))

survival_observed_3_final <- exp(rowMeans(log(survival_observed_3_combine[,-1])))

survival_observed_3_var_final <- rowMeans(survival_observed_3_var_combine[,-1]) + (1+1/n_impu)*apply(survival_observed_3_var_combine[,-1], MARGIN=1, FUN=var, na.rm=TRUE)

survival_lower3_final<-exp(log(survival_observed_3_final) - qnorm(0.975)*survival_observed_3_var_final^0.5)

survival_upper3_final<-exp(log(survival_observed_3_final) + qnorm(0.975)*survival_observed_3_var_final^0.5)

survival_comparison3 <- data.frame(survival_predicted_3_final, survival_observed_3_final,

survival_lower3_final,survival_upper3_final)

survival_comparison3$survival_upper3_final<-ifelse(survival_comparison3$survival_upper3_final>1, 1,survival_comparison3$survival_upper3_final)

survival_comparison3$underestimate<-(survival_comparison3$survival_observed_3_final-survival_comparison3$survival_predicted_3_final)/survival_comparison3$survival_observed_3_final

c3<-ggplot(data=survival_comparison3, aes(x=survival_predicted_3_final, y=survival_observed_3_final)) +

geom_line(size=1, colour="dodgerblue3")+

geom_errorbar(data=survival_comparison3, mapping=aes(x=survival_predicted_3_final, ymin=survival_lower3_final,

ymax=survival_upper3_final),

colour="dodgerblue3", size=1,alpha=0.5, linetype=1)+

geom_point(size=3, colour="dodgerblue3")+

xlim(0.7,1)+

ylim(0.7,1)+

geom_abline(intercept = 0, slope = 1,lty=2)+

labs(title="C. Calibration curve at 3 year",x="Predicted Survival Probability", y = "Observed Survival Probability")+

theme( axis.text = element_text(size = 15), axis.title=element_text(size=15),

panel.background = element_rect(fill = "white"),

plot.title = element_text(size=15),

axis.title.y = element_text(margin = margin(t = 0, r = 0.5, b = 0, l = 0, "cm")),

axis.title.x= element_text(margin = margin(t = 0.5, r = 0, b = 0, l = 0, "cm")),

plot.margin = margin(1, 1, 1, 1, "cm"),

axis.line = element_line(color='black'),

)

ggarrange(c1, c2, c3, ncol = 3, nrow = 1)

####### Sensitivity analysis: assuming missing values for HIV viral load had the same distribution as reported in the Wenzhou study #######

baselineend$misviral<-as.factor(ifelse(is.na(baselineend$viralload), 1, 0))

misviral<-subset(baselineend, select = c('id', 'misviral'))

##### ## Analyze the imputed data #####

sample_size <- nrow(baselineend)

lp_nomogram_impu <- seq(1:sample_size)

S1_nomogram <- seq(1:sample_size)

S2_nomogram <- seq(1:sample_size)

S3_nomogram <- seq(1:sample_size)

viral <- seq(1:sample_size)

CD4 <- seq(1:sample_size)

HG <- seq(1:sample_size)

# extract imputed data and calculate the predicted probability

n_impu <- 50

data_impu_0 <- vector(n_impu,mode="list")

data_impu <- vector(n_impu,mode="list")

data_impu_no <- vector(n_impu,mode="list")

data_impu_mis <- vector(n_impu,mode="list")

for (i in 1:n_impu) {

data_impu_0[[i]] <- mice::complete(imputation_50, i)

data_impu_0[[i]]$death<-as.numeric(data_impu_0[[i]]$death)-1

# transform log back

data_impu_0[[i]]$viral.load.con <- exp(data_impu_0[[i]]$logviral)

data_impu_0[[i]]$BCD4 <- exp(data_impu_0[[i]]$logCD4)

data_impu_0[[i]]$haemoglobin <- exp(data_impu_0[[i]]$loghaemoglobin)

# merge new viral

data_impu_0[[i]]<-merge(data_impu_0[[i]], misviral, by='id')

# categorize missing HIV viral load based on distribution in Wenzhou study

## subset viral without missing

data_impu_no[[i]]<-subset(data_impu_0[[i]], misviral==0)

data_impu_no[[i]]$viral.load <- ifelse((data_impu_no[[i]]$viral.load.con<200),1,

ifelse(data_impu_no[[i]]$viral.load.con>=1000,3,2))

## subset viral with missing

data_impu_mis[[i]]<-subset(data_impu_0[[i]], misviral==1)

data_impu_mis[[i]]$viral.load <- ifelse((data_impu_mis[[i]]$viral.load.con<quantile(data_impu_mis[[i]]$viral.load.con, 0.787)),1,

ifelse(data_impu_mis[[i]]$viral.load.con>=quantile(data_impu_mis[[i]]$viral.load.con, 0.817),3,2))

## rbind

data_impu[[i]]<-bind_rows(data_impu_no[[i]], data_impu_mis[[i]])

# extract viral load, CD4 and haemoglobin

viral <- data.frame(viral,data_impu[[i]]$viral.load.con)

# calculate score for viral.load

data_impu[[i]]$score_viral.load <- ifelse(data_impu[[i]]$viral.load==2,1.019669556,ifelse(data_impu[[i]]$viral.load==3,2.608969326,0))

# calculate linear predictor

data_impu[[i]]$lp_nomogram <- data_impu[[i]]$score_viral.load -0.005580907*data_impu[[i]]$BCD4 -0.005368102*data_impu[[i]]$haemoglobin

lp_nomogram_impu <- data.frame(lp_nomogram_impu,data_impu[[i]]$lp_nomogram)##

# calculate predicted survival probability

## based on formula extracted from nomogram

data_impu[[i]]$S1_nomogram <- 0.980222074^exp(data_impu[[i]]$lp_nomogram)

data_impu[[i]]$S2_nomogram <- 0.972736744^exp(data_impu[[i]]$lp_nomogram)

data_impu[[i]]$S3_nomogram <- 0.964896148^exp(data_impu[[i]]$lp_nomogram)

# add age and sex to linear predictor

## age code (<40=1, 40-59=2,>=60=3) & ###score###code gender(Men=1, Female=2)##score

data_impu[[i]]<-within.data.frame(data_impu[[i]], {

age.code<-ifelse(age<40, 1,

ifelse(age>=60,3,2))

score_age<-ifelse(age.code==2,log(1.269),

ifelse(age.code==3,log(1.798),0))

sex.code<-ifelse(gender=='Men',1,2)

score_sex<-ifelse(sex.code==2, log(0.7), 0)

})

data_impu[[i]]$lp_nomogram_age_sex <- data_impu[[i]]$lp_nomogram + data_impu[[i]]$score_age + data_impu[[i]]$score_sex

}

summary(data_impu[[1]])

nrow(data_impu[[1]])

# analyze the imputed data

calibration_slope <- 0

calibration_slope_var <- 0

c_harrel <- 0

c_harrel_var <- 0

c_harrel_age_sex <- 0

c_harrel_age_sex_var <- 0

c_harrel_diff <- 0

c_harrel_diff_var <- 0

c_time <- 0

c_time_age_sex <- 0

c_time_combine <- seq(1:20)/2

c_time_age_sex_combine <- seq(1:20)/2

IDI_combine <- seq(1:20)/2

survival_predicted_1_combine <- seq(1:10)

survival_predicted_2_combine <- seq(1:10)

survival_predicted_3_combine <- seq(1:10)

survival_observed_1_combine <- seq(1:10)

survival_observed_2_combine <- seq(1:10)

survival_observed_3_combine <- seq(1:10)

survival_observed_1_var_combine <- seq(1:10)

survival_observed_2_var_combine <- seq(1:10)

survival_observed_3_var_combine <- seq(1:10)

for (i in 1:n_impu) {

validation <- cph(Surv(time, death)~lp_nomogram, data=data_impu[[i]],x=TRUE,y=TRUE)

validation_age_sex<- cph(Surv(time, death)~lp_nomogram_age_sex, data=data_impu[[i]],x=TRUE,y=TRUE)

## Time dependent C-statistics (R package riskRegression)

c_time <- 0

c_time_age_sex <- 0

for (j in 1:20){

c_time[j] <- Score(list(validation),Surv(time,death)~1,data=data_impu[[i]],times=(0.5*j), plots="cal",metrics=c("AUC"))$AUC$score$AUC

c_time_age_sex[j] <- Score(list(validation_age_sex),Surv(time,death)~1,data=data_impu[[i]],times=(0.5*j), plots="cal",metrics=c("AUC"))$AUC$score$AUC

}

c_time_combine <- data.frame(c_time_combine,c_time)

c_time_age_sex_combine <- data.frame(c_time_age_sex_combine,c_time_age_sex)

## Calibration plot

data_impu[[i]]$group10<-cut(data_impu[[i]]$lp_nomogram, quantile(data_impu[[i]]$lp_nomogram, seq(0,1,0.1)), right=FALSE, labels=c(1:10))

data_impu[[i]]$group10[data_impu[[i]]$lp_nomogram==max(data_impu[[i]]$lp_nomogram)] <- 10

survival_predicted_1 <- 0

survival_predicted_2 <- 0

survival_predicted_3 <- 0

# 1-year predicted survival

survival_predicted_1 <- aggregate(data_impu[[i]]$S1_nomogram, list(data_impu[[i]]$group10), mean)

survival_predicted_1_combine <- data.frame(survival_predicted_1_combine,survival_predicted_1$x)

# 2-year predicted survival

survival_predicted_2 <- aggregate(data_impu[[i]]$S2_nomogram, list(data_impu[[i]]$group10), mean)

survival_predicted_2_combine <- data.frame(survival_predicted_2_combine,survival_predicted_2$x)

# 3-year predicted survival

survival_predicted_3 <- aggregate(data_impu[[i]]$S3_nomogram, list(data_impu[[i]]$group10), mean)

survival_predicted_3_combine <- data.frame(survival_predicted_3_combine,survival_predicted_3$x)

# observed survival

survival_observed_1 <- 0

survival_observed_1_var <- 0

survival_observed_2 <- 0

survival_observed_2_var <- 0

survival_observed_3 <- 0

survival_observed_3_var <- 0

for (j in 1:10) {

data_temp <- subset(data_impu[[i]],data_impu[[i]]$group10==j)

fit_calibration <- survfit(Surv(time,death) ~ 1, data=data_temp)

survival_observed_1[j] <- min(fit_calibration$surv[fit_calibration$time <= 1])

survival_observed_1_var[j] <- (tail(fit_calibration$std.err[fit_calibration$time <= 1],1))^2

survival_observed_2[j] <- min(fit_calibration$surv[fit_calibration$time <= 2])

survival_observed_2_var[j] <- (tail(fit_calibration$std.err[fit_calibration$time <= 2],1))^2

survival_observed_3[j] <- min(fit_calibration$surv[fit_calibration$time <= 3])

survival_observed_3_var[j] <- (tail(fit_calibration$std.err[fit_calibration$time <= 3],1))^2

}

survival_observed_1_combine <- data.frame(survival_observed_1_combine,survival_observed_1)

survival_observed_2_combine <- data.frame(survival_observed_2_combine,survival_observed_2)

survival_observed_3_combine <- data.frame(survival_observed_3_combine,survival_observed_3)

survival_observed_1_var_combine <- data.frame(survival_observed_1_var_combine,survival_observed_1_var)

survival_observed_2_var_combine <- data.frame(survival_observed_2_var_combine,survival_observed_2_var)

survival_observed_3_var_combine <- data.frame(survival_observed_3_var_combine,survival_observed_3_var)

}

# combine results from 50 imputation

##### line graph of linear predictor

lp_nomogram_final<-rowMeans(lp_nomogram_impu[,-1])

lp_nomogram_impu<-data.frame(seq(1:sample_size), lp_nomogram_final)

lp_nomogram_impu$risksc<-108.3333333 + 19.90914787*lp_nomogram_impu$lp_nomogram_final

summary(lp_nomogram_impu$lp_nomogram_final)

summary(lp_nomogram_impu$risksc)

lp_nomogram_impu$riskg<-as.factor(ifelse(lp_nomogram_impu$lp_nomogram_final<=0.184169968 ,"Low", "Intermediate"

))

summary(lp_nomogram_impu$riskg)

ggplot(lp_nomogram_impu, aes(x=lp_nomogram_final, y=..density..), break.x.by=0.1,) +

geom_histogram(binwidth=0.01, color="skyblue", fill="skyblue")+

scale_x_continuous(breaks = seq(-5.5,3,0.5), limits = c(-5.5,3), name='Linear predictor')+

scale_y_continuous(breaks=seq(0,1, 0.1), limits = c(0,0.8))+

theme(axis.text = element_text(size = 15), axis.title=element_text(size=15),

panel.background = element_rect(fill = "white"),

axis.line = element_line(color='black'),

axis.title.y = element_text(margin = margin(t = 0, r = 0.5, b = 0, l = 0, "cm")),

axis.title.x= element_text(margin = margin(t = 0.5, r = 0, b = 0, l = 0, "cm") ))+

geom_vline(aes(xintercept=0.184169968),linetype="dashed")+

geom_vline(aes(xintercept=2.896490986),linetype="dashed")

## time dependent C-statistics

# Wenzhou model

c_time_final <- rowMeans(c_time_combine[,-1])

c_time_combime <- data.frame(year,c_time_final)

# Wenzhou model+age+sex

c_time_age_sex_final <- rowMeans(c_time_age_sex_combine[,-1])

# then make a line chart with time as x, c_time and c_time_age_sex as y

year <- seq(1:20)/2

c_time_combime <- data.frame(year,c_time_final,c_time_age_sex_final)

c_time_combimer<- reshape2::melt(c_time_combime, id.var='year')

save(c_time_combimer, file='dependentCline.RData')

summary(c_time_combimer$value)

f1<-ggplot(c_time_combimer, aes(x=year, y=value, col=variable)) + geom_line(size=1)+geom_point(aes(shape=variable), size=3)+

labs(y = 'C-statistics',

x = "Time since ART initiation (years)")+

scale_color_manual(values=c("steelblue3", "darkred"),

name ="Model",

breaks=c("c_time_final","c_time_age_sex_final"),

labels=c("Wenzhou model", "Wenzhou model+age+sex"))+

scale_y_continuous(breaks = seq(0.4,0.9, 0.1), limits = c(0.4,0.9) )+

scale_x_continuous(breaks=seq(0,10, 1), limits = c(0,10))+

scale_shape_discrete(name ="Model",

breaks=c("c_time_final","c_time_age_sex_final"),

labels=c("Wenzhou model", "Wenzhou model+age+sex"))+

theme(legend.position=c(0.8,0.9),

legend.text = element_text(size=14),

legend.title = element_text(size=14),

legend.key=element_blank(),

legend.background=element_blank(),

axis.text = element_text(size = 15), axis.title=element_text(size=15),

panel.background = element_rect(fill = "white"),

axis.line = element_line(color='black'),

axis.title.y = element_text(margin = margin(t = 0, r = 0.5, b = 0, l = 0, "cm")),

axis.title.x= element_text(margin = margin(t = 0.5, r = 0, b = 0, l = 0, "cm")),

plot.margin = margin(1, 1, 1, 1, "cm"))

# calibration plot

# 1-year

survival_predicted_1_final <- exp(rowMeans(log(survival_predicted_1_combine[,-1])))

survival_observed_1_final <- exp(rowMeans(log(survival_observed_1_combine[,-1])))

survival_observed_1_var_final <- rowMeans(survival_observed_1_var_combine[,-1]) + (1+1/n_impu)*apply(survival_observed_1_var_combine[,-1], MARGIN=1, FUN=var, na.rm=TRUE)

survival_lower1_final<-exp(log(survival_observed_1_final) - qnorm(0.975)*survival_observed_1_var_final^0.5)

survival_upper1_final<-exp(log(survival_observed_1_final) + qnorm(0.975)*survival_observed_1_var_final^0.5)

survival_comparison1 <- data.frame(survival_predicted_1_final, survival_observed_1_final,

survival_lower1_final,survival_upper1_final)

survival_comparison1$survival_upper1_final<-ifelse(survival_comparison1$survival_upper1_final>1, 1,survival_comparison1$survival_upper1_final)

survival_comparison1$underestimate<-(survival_comparison1$survival_observed_1_final-survival_comparison1$survival_predicted_1_final)/survival_comparison1$survival_observed_1_final

c1<-ggplot(data=survival_comparison1, aes(x=survival_predicted_1_final, y=survival_observed_1_final)) +

geom_line(size=1, colour="dodgerblue3")+

geom_errorbar(data=survival_comparison1, mapping=aes(x=survival_predicted_1_final, ymin=survival_lower1_final,

ymax=survival_upper1_final),

colour="dodgerblue3", size=1,alpha=0.5, linetype=1)+

geom_point(size=3, colour="dodgerblue3")+

xlim(0.7,1)+

ylim(0.7,1)+

geom_abline(intercept = 0, slope = 1,lty=2)+

labs(title="A. Calibration curve at 1 year",x="Predicted Survival Probability", y = "Observed Survival Probability")+

theme( axis.text = element_text(size = 15), axis.title=element_text(size=15),

panel.background = element_rect(fill = "white"),

plot.title = element_text(size=15),

axis.title.y = element_text(margin = margin(t = 0, r = 0.5, b = 0, l = 0, "cm")),

axis.title.x= element_text(margin = margin(t = 0.5, r = 0, b = 0, l = 0, "cm")),

plot.margin = margin(1, 1, 1, 1, "cm"),

axis.line = element_line(color='black'),

)

(mean(survival_comparison1$survival_observed_1_final)-mean(survival_comparison1$survival_predicted_1_final))*100

(mean(survival_comparison2$survival_observed_2_final)-mean(survival_comparison2$survival_predicted_2_final))*100

(mean(survival_comparison3$survival_observed_3_final)-mean(survival_comparison3$survival_predicted_3_final))*100

# 2-year

survival_predicted_2_final <- exp(rowMeans(log(survival_predicted_2_combine[,-1])))

survival_observed_2_final <- exp(rowMeans(log(survival_observed_2_combine[,-1])))

survival_observed_2_var_final <- rowMeans(survival_observed_2_var_combine[,-1]) + (1+1/n_impu)*apply(survival_observed_2_var_combine[,-1], MARGIN=1, FUN=var, na.rm=TRUE)

survival_lower2_final<-exp(log(survival_observed_2_final) - qnorm(0.975)*survival_observed_2_var_final^0.5)

survival_upper2_final<-exp(log(survival_observed_2_final) + qnorm(0.975)*survival_observed_2_var_final^0.5)

survival_comparison2 <- data.frame(survival_predicted_2_final, survival_observed_2_final,

survival_lower2_final,survival_upper2_final)

survival_comparison2$survival_upper2_final<-ifelse(survival_comparison2$survival_upper2_final>1, 1,survival_comparison2$survival_upper2_final)

survival_comparison2$underestimate<-(survival_comparison2$survival_observed_2_final-survival_comparison2$survival_predicted_2_final)/survival_comparison2$survival_observed_2_final

c2<-ggplot(data=survival_comparison2, aes(x=survival_predicted_2_final, y=survival_observed_2_final)) +

geom_line(size=1, colour="dodgerblue3")+

geom_errorbar(data=survival_comparison2, mapping=aes(x=survival_predicted_2_final, ymin=survival_lower2_final,

ymax=survival_upper2_final),

colour="dodgerblue3", size=1,alpha=0.5, linetype=1)+

geom_point(size=3, colour="dodgerblue3")+

xlim(0.7,1)+

ylim(0.7,1)+

geom_abline(intercept = 0, slope = 1,lty=2)+

labs(title="B. Calibration curve at 2 year",x="Predicted Survival Probability", y = "Observed Survival Probability")+

theme( axis.text = element_text(size = 15), axis.title=element_text(size=15),

panel.background = element_rect(fill = "white"),

plot.title = element_text(size=15),

axis.title.y = element_text(margin = margin(t = 0, r = 0.5, b = 0, l = 0, "cm")),

axis.title.x= element_text(margin = margin(t = 0.5, r = 0, b = 0, l = 0, "cm")),

plot.margin = margin(1, 1, 1, 1, "cm"),

axis.line = element_line(color='black'),

)

# 3-year

survival_predicted_3_final <- exp(rowMeans(log(survival_predicted_3_combine[,-1])))

survival_observed_3_final <- exp(rowMeans(log(survival_observed_3_combine[,-1])))

survival_observed_3_var_final <- rowMeans(survival_observed_3_var_combine[,-1]) + (1+1/n_impu)*apply(survival_observed_3_var_combine[,-1], MARGIN=1, FUN=var, na.rm=TRUE)

survival_lower3_final<-exp(log(survival_observed_3_final) - qnorm(0.975)*survival_observed_3_var_final^0.5)

survival_upper3_final<-exp(log(survival_observed_3_final) + qnorm(0.975)*survival_observed_3_var_final^0.5)

survival_comparison3 <- data.frame(survival_predicted_3_final, survival_observed_3_final,

survival_lower3_final,survival_upper3_final)

survival_comparison3$survival_upper3_final<-ifelse(survival_comparison3$survival_upper3_final>1, 1,survival_comparison3$survival_upper3_final)

survival_comparison3$underestimate<-(survival_comparison3$survival_observed_3_final-survival_comparison3$survival_predicted_3_final)/survival_comparison3$survival_observed_3_final

c3<-ggplot(data=survival_comparison3, aes(x=survival_predicted_3_final, y=survival_observed_3_final)) +

geom_line(size=1, colour="dodgerblue3")+

geom_errorbar(data=survival_comparison3, mapping=aes(x=survival_predicted_3_final, ymin=survival_lower3_final,

ymax=survival_upper3_final),

colour="dodgerblue3", size=1,alpha=0.5, linetype=1)+

geom_point(size=3, colour="dodgerblue3")+

xlim(0.7,1)+

ylim(0.7,1)+

geom_abline(intercept = 0, slope = 1,lty=2)+

labs(title="C. Calibration curve at 3 year",x="Predicted Survival Probability", y = "Observed Survival Probability")+

theme( axis.text = element_text(size = 15), axis.title=element_text(size=15),

panel.background = element_rect(fill = "white"),

plot.title = element_text(size=15),

axis.title.y = element_text(margin = margin(t = 0, r = 0.5, b = 0, l = 0, "cm")),

axis.title.x= element_text(margin = margin(t = 0.5, r = 0, b = 0, l = 0, "cm")),

plot.margin = margin(1, 1, 1, 1, "cm"),

axis.line = element_line(color='black'),

)

ggarrange(c1, c2, c3, ncol = 3, nrow = 1)
